# The morphological spectrum of Castleman disease and related disorders: a report from the Lymphoma Workshop of the 22nd Meeting of the European Association of Hematopathology

**DOI:** 10.1007/s00428-025-04171-w

**Published:** 2025-07-12

**Authors:** Gorana Gasljevic, Arturo Bonometti, Ioannis Anagnostopoulos, Olga Balaguè, Michiel Van den Brand, James R. Cook, Camille Laurent, Maurilio Ponzoni, Leticia Quintanilla-Martinez, Birgitta Sander, Stefan Dirnhofer

**Affiliations:** 1 Department of Pathology, Institute of Oncology Ljubljana, Zaloška Cesta 2, 1000, Ljubljana, Slovenia; 2 Medical Faculty, University of Maribor, Taborska cesta 8, Maribor, Slovenia; 3 Department of Biomedical Sciences, Humanitas University, Via Rita Levi Montalcini 4, Pieve Emanuele, Milan, 20090 Italy; 4 Department of Pathology Unit, Humanitas Clinical and Research Hospital, Via Manzoni 56, Rozzano, Milan, 20089 Italy; 5 Institute of Pathology, Julius-Maximilians-Universität Würzburg, Würzburg, Germany; 6 Hospital Clinic, Pathology Department, Barcelona, Spain; 7 Department of Pathology, Radboud University Medical Center, Nijmegen, The Netherlands; 8 Pathology-DNA location Rijnstate Hospital, Arnhem, The Netherlands; 9 Pathology and Laboratory Medicine Institute, Cleveland Clinic, Cleveland, OH US; 10 Department of Pathology, Institut Universitaire du Cancer-Oncopole de Toulouse CHU Toulouse, Toulouse, France; 11 Ateneo Vita-Salute San Raffaele Milan and Pathology Unit, IRCCS San Raffaele Scientific Institute, Milan, Italy; 12 Institute for Pathology and Neuropathology, Eberhard-Karls-University of Tübingen and Comprehensive Cancer Center, University Hospital Tübingen, Tübingen, Germany; 13 Cluster of Excellence iFIT (EXC2180) “Image-guided and Functionally Instructed Tumor Therapies” Eberhard-Karls-University of Tübingen, Tübingen, Germany; 14 Department of Laboratory Medicine, Division of Pathology, Karolinska Institutet and Karolinska University Hospital, Stockholm, Sweden; 15 Institute of Medical Genetics and Pathology, University Hospital Basel, University of Basel, Basel, Switzerland

**Keywords:** Castleman disease, Idiopathic multicentric Castleman disease, TAFRO syndrome, POEMS syndrome, KSHV/HHV8-associated lymphoproliferative disorders, CD-mimickers

## Abstract

Castleman disease (CD) is an intriguing and complicated group of local and systemic disorders mainly affecting lymph nodes with heterogeneous presentation and therapeutic needs. These disorders were the topic of Session 1 of the Lymphoma Workshop at the 2024 EA4HP in Dubrovnik, Croatia. In this report, we summarize the features of the 85 submitted cases and review the differential diagnosis, pitfalls, and advances for all CD subtypes. Specifically, the molecular landscape of unicentric CD and its relationship with follicular dendritic cell proliferations and indolent T-lymphoblastic proliferation will be discussed. The spectrum of idiopathic multicentric CD (MCD), TAFRO syndrome, as well as the clinical and histopathological peculiarities of POEMS-CD, is reviewed. Cases of Kaposi sarcoma-associated herpesvirus/human herpesvirus 8 (KSHV/HHV8) + MCD were the most complicated and well demonstrated the difficulties and overlaps in the differential diagnosis of KSHV/HHV8 + lymphoproliferative disorders. Finally, the important topic of CD mimickers will be addressed, demonstrating how the integration of clinical, laboratory, histopathological, and molecular data is mandatory to confirm a diagnosis of CD and how to distinguish it from the many neoplastic, autoimmune, and infective mimickers.

**Supplementary Information:**

The online version contains supplementary material available at 10.1007/s00428-025-04171-w.

## Introduction

Castleman disease (CD) represents a complex group of at least four rare lymphoproliferative disorders morphologically defined by Benjamin Castleman in cases of unicentric hyaline-vascular CD (HV-CD) [1]. Although the use of the term “disease” suggests that the entity has a known cause, defined pathophysiology, and consistent clinical and/or pathological findings, this is still not the case, and it may be better to consider CD as a group of clinical syndromes linked to multiple etiologies. As the spectrum of non-neoplastic diseases that can mimic such a morphology expanded, so has the broader histological definition of CD. CD typically involves lymph nodes (LNs), while it may also rarely affect extranodal sites (e.g., spleen or thymus) [2].

**Fig. 1:**
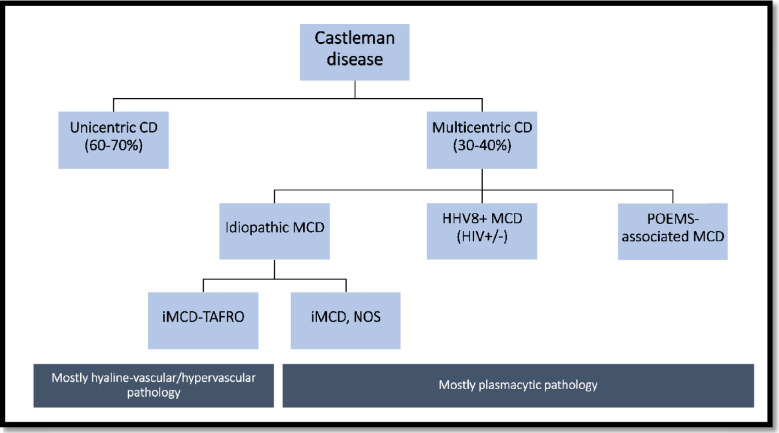
Clinical classification of CD, associated syndromes, and histological features

According to the number of involved LNs, CD is clinically divided into unicentric (UCD, i.e., one LN station involved) and multicentric (MCD, i.e., involving two or more lymph node groups) (Fig. 1). MCD is associated with clinical and laboratory stigmata of systemic inflammation and is further divided into three clinical subsets: (1) MCD associated with KSHV/HHV8 infection (MCD-HHV8); (2) MCD associated with plasma cell disorders and neurological and endocrinological symptoms (MCD-POEMS); and (3) MCD patients without known etiology, hence categorized as idiopathic MCD (iMCD). iMCD can be further subclassified into iMCD-thrombocytopenia, ascites, reticulin fibrosis/renal dysfunction, organomegaly (iMCD-TAFRO), or iMCD-not otherwise specified (iMCD-NOS).

CD is also histopathologically heterogeneous. Indeed, CD may fall between two ends of a spectrum of changes, known as hyaline vascular (HV) and plasmacytic (PV) variants, with cases showing both HV and PV features, known as mixed variant (MV) [3, 4]. In HV, LNs are enlarged with a fibrotic capsule and compressed and obliterated sinuses. Follicles are increased in number and size and have germinal centers (GC) with lymphocyte depletion and increased follicular dendritic cells (FDC). Follicles may contain more than one GC surrounded by a single mantle layer (known as “twinning/budding”). The mantle zones are expanded and constituted by concentrically arranged layers of lymphocytes (“onion-skinning”). Nodal vascularity is increased and radially orientated vessels may penetrate GCs (“lollipop-like” image). Interfollicular areas contain mainly small lymphocytes and numerous high endothelial venules, fibrosis, and clusters of plasmacytoid dendritic cells (PDC). Some cases may display an interfollicular overgrowth of a variety of stromal cells (fibroblastic, myofibroblastic, and/or histiocytic-derived stromal cells) known as stroma-rich variant (SRV) [4, 5].

In PV and MV, the LNs are usually smaller and characterized by follicular hyperplasia with follicular regression and expansion of interfollicular areas by aggregates and sheets of mature plasma cells. Features of HV may be present, but are much less evident, and blood vessels are variably prominent [4].

In both UCD and MCD, the nodal microenvironmental composition seems to be involved in the pathogenesis of the disease, and the production of the typical cytokine milieu is found in most subsets. Indeed, some studies demonstrated the production of interleukin 6 (IL-6) by endothelial cells, and of vascular endothelial growth factor (VEGF) by FDC and resident macrophages, while others highlighted an imbalance in T-cell subsets across CD subtypes [6–9].

Given the diagnostic difficulties often encountered in CD, an excisional LN biopsy is mandatory for the diagnosis, together with the multidisciplinary integration of clinical and laboratory data and the exclusion of potential mimickers.

The topic of the Lymphoma Workshop (LYWS) Session 1 at the 2024 European Association for Hematopathology (EA4HP) and Society for Hematopathology (SH) meeting in Dubrovnik, Croatia, was dedicated to the morphological spectrum of CD and its mimickers. Altogether, 85 cases were submitted representing the many challenges in the diagnosis of CD and CD-related disorders. The cases were divided into six thematic groups to illustrate morphological overlap and diagnostic and biological features of these diseases:

1. UCD
2. without atypical FDC proliferation (AFDCP)/FDC sarcoma (FDCS)
3. with proliferation of monoclonal plasma cells
4. with AFDCP/FDCS
5. with indolent T-lymphoblastic proliferation (iT-LPB)
6. iMCD-NOS
7. iMCD-TAFRO
8. MCD-POEMS
9. MCD-HHV8 and related disorders
10. CD-mimickers
  - lymphomas
  - reactive lymphadenopathies
  - autoimmune/autoinflammatory disorders
  - IgG4-related disease
  - Other

A summary of the findings and conclusions arising from the discussion of these cases is presented in this report with an overview given in Table 1.

**Table 1:** Overview of all cases submitted and studied for the Lymphoma Workshop (LYWS) Session 1 of the 2024 European Association for Hematopathology (EA4HP) and Society for Hematopathology (SH)

Category	Histological type	Number of WS cases	Unusual features
**UCD with no associated FDC proliferation**	Hyaline vascular	10	3/10 cases stroma-rich 1/10 case with systemic symptoms 1/10 case associated with thymoma AB 1/10 case with probable *SRP72* germline mutation 1/10 case with extensive ossification 2/10 cases with associated iT- LBP
	Mixed type	1	Stroma rich
**UCD with proliferation of monoclonal plasma cells**	Hyaline vascular	1	Lambda predominance of unknown significance
	Mixed type	1	Lambda restriction
	Plasma cell type	2	IgA lambda restriction in both 1/2 loss of* D13S319*
**UCD associated with atypical FDC proliferation**	Hyaline vascular	2	1/2 case loss of FDC markers 1/2 case with associated iT-LBP
**UCD associated with FDC sarcoma**	Hyaline vascular	7	2/7 cases with associated iT-LBP
**iMCD-NOS**	Mixed/plasma cell type	5	1/5 case borderline clinicopathological features between IgG4-RD and iMCD- NOS 1/5 case overlapping of iMCD-NOS with TAFRO and POEMS syndrome 3/5 cases increased IgG4 plasma cells and increased serum IgG4 1/5 cases progression to cHL
**iMCD-TAFRO and TAFRO syndrome**	Mixed/plasma cell type	3	1/4 case demyelinating polyneuropathy
	Hypervascular type (“hyper-V”)	2	
	No LN biopsy	1	Masaki’s criteria
			In all cases, BM changes: Mgc hyperplasia and RF grade I or II
**POEMS-associated MCD**	Mixed/plasma cell type	4	1/4 case after Covid-19 vaccination 1/4 case after Covid-19 infection
			In all cases, BM plasmacytosis 5–10% 3/4 cases IgAλ restricted 1/4 cases Mgc hyperplasia
**HHV8-associated MCD**	KSHV/HHV8-associated MCD	25	4/25 cases associated with plasmablasts’ aggregates (one case mimicking GLPD and in one case IgH rearrangement) 9/25 cases with associated Kaposi sarcoma 1/25 case with splenic involvement 1/25 case with associated secondary HLH 7/25 cases with associated EBV reactivation 1/25 case development of subsequent PEL, EBV negative 1/25 case associated iT-LBP 1/25 case with concurrent histoplasmosis 11/23 cases HIV negative
	KSHV/HHV8-positive GLPD with CD-like features	1	
	KSHV/HHV8 + DLBCL vs EC-PEL	3	1/3 case proliferation of highly atypical KSHV/HHV8 + cells 1/3 case associated Kaposi sarcoma
	HHV8 + DLBCL	1	
**CD-mimickers**	FL with HV-CD features	3	1/3 case *BCL2*-R-negative CD23 + FL 1/3 case long-lasting SAPHO syndrome
	MZL with CD-HV features	2	1/2 case EMZL NMZL increased IgG4 plasma cells
	cHL with CD-mixed type features	1	Thymic cHL
	Sarcoidosis with CD-like features and AA-type amyloidosis	1	
	EBV reactive hyperplasia in immunocompetent person with CD-like features	1	
	Schnitzler syndrome with CD-like features	1	
	IgG4-related LAD with CD-like features (different patterns)	5	5/5 cases expanded interfollicular and/or peri- follicular polytypic plasma cell population with IgG4/IgG > 40% and IgG4 + plasma cells > 400/mm^2^ 3/5 cases increased serum IgG4
	FH with some CD-like features	1	IEI to be considered (young child with generalized LAD and splenomegaly)
**Other**	KS in isolated LN of immunocompetent patient	1	

*UCD* unicentric Castleman disease, *FDC* follicular dendritic cells, *iT-LBP* indolent T-lymphoblastic proliferation, *IgG4-RD* IgG4-related disease, *LN* lymph node, *BM* bone marrow, *GLPD* germinotropic lymphoproliferative disorder, *SAPHO* synovitis, arthritis, pustulosis, hyperostosis, osteitis, *MZL* marginal zone lymphoma, *EMZL* extranodal marginal zone lymphoma, *NMZL* nodal marginal zone lymphoma, *cHL* classical Hodgkin’s lymphoma, *IEI* inborn error of immunity

## Unicentric Castleman disease

UCD affects one or more locoregional LNs and is the most common clinical subtype of CD (60–70%). It is typically asymptomatic and up to 80–90% of cases present with HV histology. Despite large, localized lymphadenopathy (LAD) or extranodal masses that may reach significant size, surgical excision is curative in most cases [10]. The pathogenesis of UCD is not fully understood. Still, it may at least partially be the consequence of the clonal proliferation of stromal cells including FDC. Of interest, one study showed that around 10–20% of UCD cases harbor gain-of-function somatic mutation in the kinase domain of the *PDGFRB* gene, probably in stromal cells [11]. In addition, mutations of other genes of the IL-signaling pathway and MAPK pathway (*RAS* family,* FGFR3*,* JAK2/3*,* NF1*…) may be identified [9, 12, 13]. Recent transcriptome data showed an upregulation of the transcripts linked to complement and coagulation cascade, collagen fiber organization, S1P-S1P3 pathway, and VEGFR signaling in UCD cases [9, 14]. An overview of the reported genetic alterations in UCD is given in Supplementary Table 1.

Patients with UCD harbor an increased risk of developing paraneoplastic pemphigus, bronchiolitis obliterans, lymphoma, or AA-amyloidosis. A further peculiarity of UCD is its association with FDC dysplasia and the development of follicular dendritic cell sarcoma (FDCS).

Altogether, 24 cases of UCD were submitted (Supplementary Table WS-1), 20 HV, and 4 MV/PV. Nine cases were associated with AFDCP/FDCS.

### UCD without AFDCP/FDCS

Three cases (LYWS-235 submitted by V. Parecki, LYWS-245 by R. Orellana-Fernandez, LYWS-448 by T. Guchash) showed typical UCD-HV features, while LYWS-251 submitted by J. Reszec-Gielazyn represented a rare case of UCD-HV with systemic symptoms and abnormal laboratory values.

**Fig. 2:**
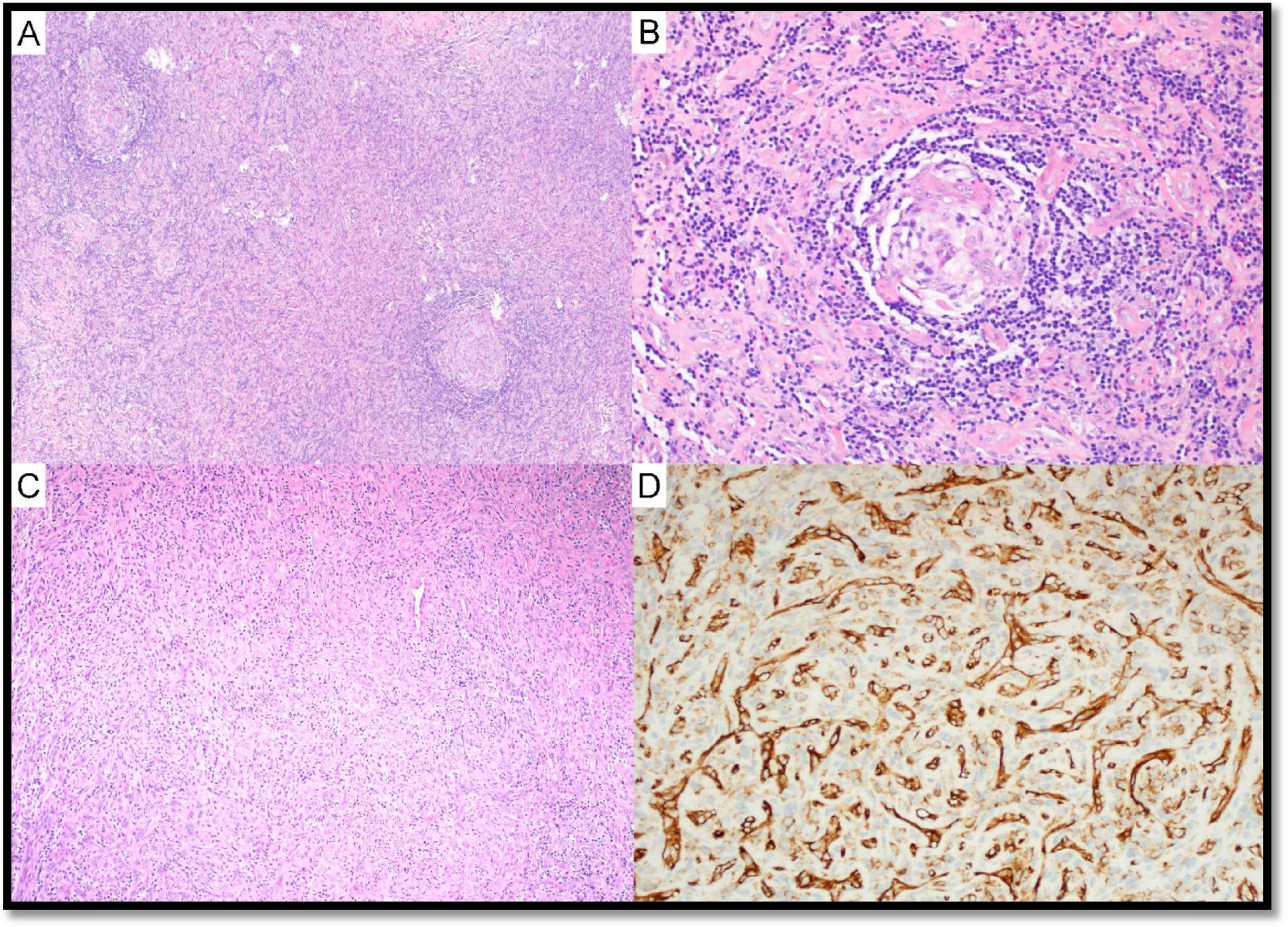
Stroma-rich variant of HV-CD. Case LyWS-101. Courtesy of R. King. **A**, **B** H&E; small follicles with regressively transformed germinal centers, concentric mantle zones, and numerous interfollicular hyalinizing small vessels that occasionally penetrate germinal centers. **C** Interfollicular stroma replaced by proliferation of hyalinized small vessels and abundant spindled, fibrous stroma. **D** CD31 highlights the density of small vessels in the stroma

Three cases of UCD-stromal rich variant (UCD-SRV) were submitted (LYWS-101 presented by R. King, (Fig. 2), LYWS-199 by N. Tabish, LYWS-224 by M. Movassaghi). This rare variant tends to occur in the adult population and usually involves abdominal LNs [5]. UCD-SRV represents a diagnostic challenge due to its rarity, varied stromal cell composition, and thus differential diagnosis with low-grade mesenchymal neoplasms (of fibrohistiocytic, muscular, and vascular origin) and FDCS. By definition, the area occupied by the interfollicular stromal proliferation is larger than the follicular one [15]. Patients with UCD-SRV often suffer from paraneoplastic pemphigus, which is associated with a poor prognosis [16].

In case LYWS-426 submitted by I. Xagoraris, an extensive ossification in the stromal component was present. Heterotopic ossification is a very rarely described phenomenon in HV-CD and can lead to a difficult radiological differential diagnosis as it may mimic both benign and malignant disorders of the neck, chest, abdomen, and pelvis [17–19].

Case LYWS-217 submitted by D. Alquaidy depicted the exceptional association of UCD with thymoma [20]. CD was described as involving the thymus in only 11 cases so far, the majority being UCD of different morphological variants [21]. Thymic CD can be associated with myasthenia gravis. It is morphologically easily distinct from thymoma, including the micronodular variant of spindle cell thymoma with prominent B-cell lymphoid hyperplasia [22]. The mainstay of therapy is thymectomy.

Case LYWS-318 submitted by J. Sidhu was a case of UCD-HV bearing a *SRP72_R324C* variant with a variant allele frequency of 51.5% suspicious for a germline mutation. Of interest, mutations in *SRP72* contribute to the development of familial MDS and AML [23].

Case LyWS-159 submitted by S. Li was UCD-MV, which progressed into CD20 +/CD5 −/CD10 − B-cell lymphoma (NHL), not further specified. The development of classic Hodgkin lymphoma (cHL) or B-cell or T-cell lymphoma can be observed in the course of CD, often involving different lymph nodes [24, 25]. HL and NHL are more often associated with CD-PV and HV-CD, respectively [26, 27]. The precise relationship between UCD, HL, and NHLs is still unclear; the risk of subsequent lymphoma is low.

### UCD with proliferation of monoclonal plasma cells

Four submitted cases featured plasma cells with λ-light chains predominance/restriction, three of them were PV/MV type, and one HV type (LYWS-53 submitted by E. Frye-Naharro, LYWS-185 by J. C. Harris, LYWS-261 by O. C. Eren, and LYWS-362 by R. Gulati). The clinical as well as prognostic significance of this finding is unclear. Plasma cell expansion is typical of PV-CD and is mostly observed in MCD cases. Plasma cells in UCD are generally polytypic; however, occasionally, they can be monotypic and λ-restricted [28–31]. Before rendering a diagnosis of UCD with monotypic plasma cells, POEMS syndrome, lymphoplasmacytic lymphoma (LPL), marginal zone lymphoma (MZL), and plasmacytoma should be carefully excluded. In case LYWS-53, cytogenetic analysis found a loss of *D13S319*, a recurrent variant in lymphoid and plasma cell disorders [32, 33]. Extraosseous involvement by a plasma cell neoplasm was ruled out by a bone marrow biopsy examination. By flow cytometry, plasma cells were highly skewed towards lambda light chain restriction. Mutations for *BIRC3*, *CXCR4*, *KLF2*, and *MYD88* were negative. Cases of CD associated with either bone or nodal plasmacytoma (out of POEMS syndrome) have been published [30, 33, 34]. Radaszkiewicz et al. described 7/18 patients with CD-PV having a monoclonal plasma cell population, five IgG-λ, and two IgA-λ type [31, 34, 35]. In none of these cases, extranodal manifestations of plasmacytoma have been found, and only 2/7 patients exhibited paraproteinaemia corresponding to the Ig type of the proliferated plasma cells. The authors suggested that the monoclonal variant of CD-PV may be a nodal type of monoclonal gammopathy [31].

### UCD associated with AFDCP/FDCS

**Fig. 3:**
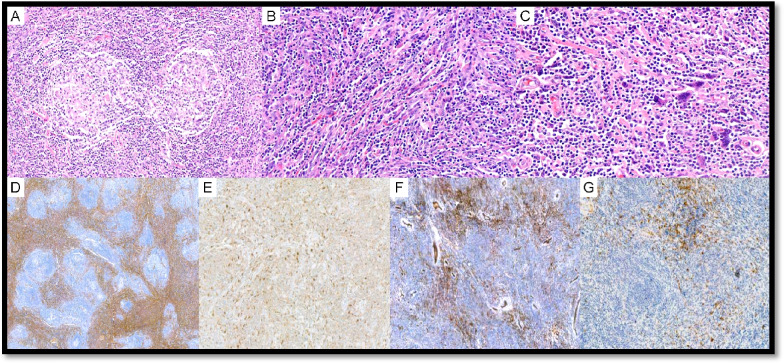
Unicentric Castleman disease with atypical stromal cell proliferation. Case LyWS-369. Courtesy of J. Bosch-Schips. **A** Hyaline vascular features: extensive proliferation of high endothelial venules with perivascular hyalinization, twinning of mantle zones and atretic germinal centers. **B** Prominent interfollicular stromal spindle cell proliferation. **C** Focal moderate-to-marked dysplastic features in stromal spindle cell proliferation. **D** Interfollicular spindle cells are positive for SMA. Interfollicular spindle cells are weakly positive for **E** CD68, **F** CD31, and **G** EMA

UCD with AFDCP was diagnosed in two cases (LYWS-271 submitted by L. D. Yuen and LYWS-455 by A. Green; Supplemental Table 2). In case LYWS-455, dysplastic CD21 + FDC in the background of HV-CD showed loss of other FDC markers but not *bona fide* sarcoma. LYWS-369 submitted by J. Bosch-Schips (Fig. 3) displayed a unique prominent interfollicular spindle cell proliferation with no obvious follicle effacement. These cells were slightly atypical and positive for SMA; focally positive for CD68, CD31, EMA, and p53; and negative for CKAE1/AE3, S100p, Desmin, CD21, CXCL13, CD23, and D2-40.

**Fig. 4:**
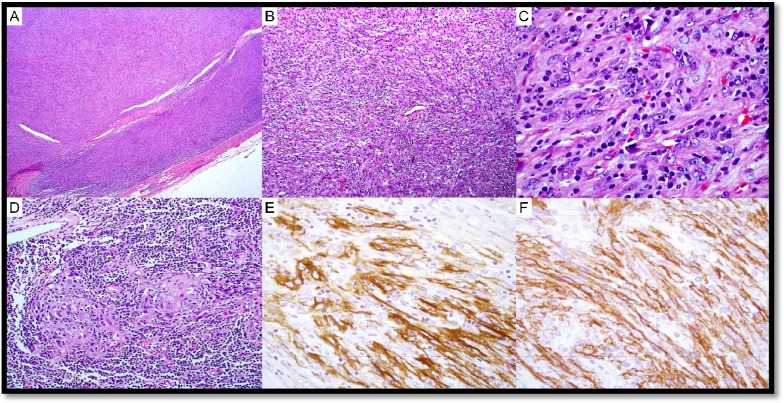
Follicular dendritic cell sarcoma associated with Castleman disease. Case LyWS-73. Courtesy of S-B. Ng. **A**, **B** Tumour composed of spindle to ovoid cells in sheets, fascicles, and storiform whorls. **C** Neoplastic cells with dispersed chromatin and small nucleoli. Scattered lymphocytes and plasma cells. **D** Proliferation of FDCs in residual follicles with hyperplastic/dysplastic features. Tumor cells are **E** CD21 and **F** CD35 positive

UCD-HV was associated with typical FDCS in 6 cases (LYWS-73 presented by S. Ng (Fig. 4), LYWS-147 submitted by Z. Hossein-Zadeh, LYWS-155 by A. Evans, LYWS-205 by X. N. Jiang, LYWS-260 by O. C. Eren, LYWS-341 by T. Tousseyn) (Supplementary Table WS-2). FDCS is a rare, low- to intermediate-grade mesenchymal neoplasm, composed of a proliferation of spindled to ovoid cells expressing FDC markers. FDCS seems to arise from ubiquitous perivascular precursors expressing PDGFRβ [36–38]. Approximately 60% of FDCS occurs in extranodal sites, and 10–20% are associated with prior or concurrent UCD-HV [39–43]. As FDC are typically located in GC, the presence of atypia (dysplasia) in FDC occurring outside the follicles is highly suggestive of transformation to FDCS [44]. A hyperplasia–dysplasia–neoplasia model of FDC proliferation has been proposed as the link between UCD-HV and FDCS and was thought to be supported by the EGFR overexpression in dysplastic FDC [45, 46]. Nevertheless, EGFR is also expressed in normal FDC, and no correlation was found between EGFR protein expression and EGFR copy number status [39, 46]. EGFR protein expression in FDCS might be a consequence of ligand-dependent activation by cognate ligands produced by the tumor microenvironment, which might influence the proliferation of FDCS cells but does not represent per se a proper oncogenic mechanism [46].

The molecular landscape of FDCS includes complex karyotype and recurrent alterations in NF-κB pathway genes and in tumor suppression genes [47–50]. Frigola et al. emphasized the role of *MYC* and *TP53* alterations in FDCS pathogenesis, while Lorenzi et al. unveiled alterations on homologous recombination DNA damage repair-related genes in 70% of FDCS cases [51, 52].

### UCD associated with indolent T-lymphoblastic proliferation (iT-LBP)

**Fig. 5:**
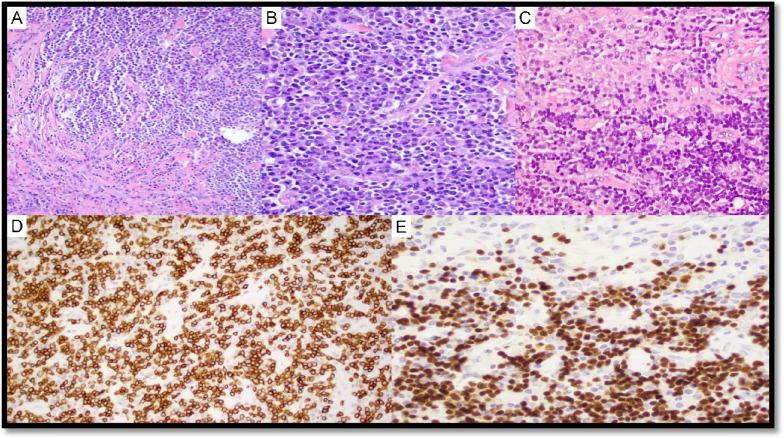
Castleman disease, mixed hyaline vascular variant and plasma cell variant, stroma-rich with iT-LBP. Case LyWS-159. Courtesy of J. Li. **A** Plasma cell and stroma-rich area. **B** Plasma cell-rich area. **C** Blastoid small lymphocytic clusters positive for **D** CD3 and **E** TdT

In six UCD cases (with or without AFDCP/FDCS), an iT-LPB was present (LYWS-73 submitted by S. Ng, LYWS-101 by R. King, LYWS-155 by A. Evans, LYWS-159 by S. Li (Fig. 5), LYWS-245 by R. Orellana-Fernandez, LYWS-369 by J. Bosch-Schip). iT-LPB is defined as an extrathymic, non-clonal expansion of T-lymphoblasts occurring alone or in association with other disorders. Associated conditions especially include CD, FDCS, and nodal T-follicular helper cell lymphomas, but also acinic cell carcinoma and hepatocellular carcinoma [53–57]. In comparison to T-lymphoblastic lymphoma (T-LBL), iT-LBPs lack significant morphologic atypia, tissue destruction, aberrant immunophenotype, and monoclonality and do not involve bone marrow or mediastinum. This topic is covered in the report of Session 5, “exploring the boundaries between neoplastic and reactive lymphoproliferations: lymphoid neoplasms with indolent behaviour and clonal lymphoproliferations.”

## iMCD-NOS

iMCD-NOS is characterized by multiple LAD and systemic inflammatory symptoms caused by hypercytokinemia (e.g., increased IL-6, VEGF, IL-2, TNFα, IL-10, and CXCL13) due to different mechanisms such as autoimmune, autoinflammatory, neoplastic, or infectious [7, 58]. A recent study of virome capture sequencing did not identify active viral infection in non-HHV8 CD (UCD and MCD) ruling out a possible viral etiology, but not a possible indirect role of viral infection [59].

iMCD-NOS accounts for 30–50% of all MCD cases and typically affects patients in the fourth and fifth decades of life, with males being more frequently affected [60]. The clinical presentation varies substantially. Some patients experience only mild constitutional symptoms while others present with cytokine storm and lethal organ failure [10, 58]. The diagnosis of iMCD-NOS is challenging, as a significant clinical, histologic, and immunologic overlap exists with different autoimmune, malignant, and infective conditions. For this reason, the Castleman Disease Collaborative Network (CDCN) recently established evidence-based criteria for the diagnosis of KSHV/HHV8-negative iMCD (Supplementary Table 2). This work was the first to underline the importance of categorizing the histopathological changes in MCD (i.e., HV and PV) and introducing a grading system. It is based on grading germinal center (GC) regression, FDC prominence, vascularity, GC hyperplasia, and plasmacytosis with a four-tiered scale (0–3) [3]. Moreover, the term “hypervascular morphology” (HyV) was introduced for HV of iMCD cases, especially for patients with TAFRO syndrome typically showing HyV histopathology. Despite these efforts, the evidence for the use of histopathologic subtype to guide the treatment of iMCD is so far insufficient [2, 3].

**Fig. 6:**
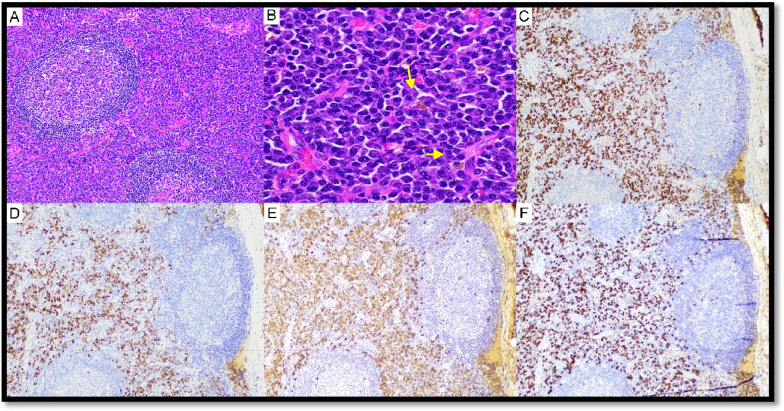
Idiopathic multicentric Castleman disease with massive infiltration of IgG4-positive plasma cells. Case LyWS-20. Courtesy of K. Karube. **A** Regressed follicles and expanded paracortex. **B** Diffuse sheets of plasma cells. Hemosiderin deposition is evident (arrows). No eosinophil infiltration. **C** IgG and **D** IgG4—IgG4/IgG ratio is about 50%. **E** Kappa light chains and **F** Lambda light chains—no light chain restriction

Five cases of iMCD-NOS were submitted to the workshop (Supplementary Table WS-3). Case LYWS-56 submitted by C. Sy showed overlapping features between iMCD-NOS and iMCD-TAFRO. Case LYWS-439 submitted by K. Horvat-Pavlov represented a typical iMCD-NOS case. LYWS-20 presented by K. Karube (Fig. 6), LYWS-71 by J. T. Kelley, and LYWS-402 by M. Meignin were cases of iMCD-NOS associated with increased IgG4 + plasma cells and elevated serum levels of IgG4. Such cases showed overlapping clinicopathological features between IgG4-related diseases (IgG4-RD) and iMCD-NOS. The diagnosis of iMCD-NOS was favored over IgG4-RD based on the clinical, laboratory, and histological findings (i.e., diffuse nodal plasmacytosis, presence of hemosiderin-laden histiocytes, and lack of eosinophils. Case LYWS-71 was diagnosed with iMCD-NOS-PV, developing a cHL 10 months later.

## iMCD-TAFRO

TAFRO syndrome is a systemic disease characterized by thrombocytopenia, anasarca, myelofibrosis, renal dysfunction, and organomegaly that may be associated with iMCD (i.e., iMCD-TAFRO). Despite iMCD-TAFRO and iMCD-NOS share their pathogenesis (hypercytokinemia and IL-6 upregulation), their clinical behavior is different and their exact relationship is still undefined [61, 62]. While iMCD-NOS generally pursues a chronic course, iMCD-TAFRO patients show an acute/subacute onset with a rapid worsening of the patient’s condition [63].

**Fig. 7:**
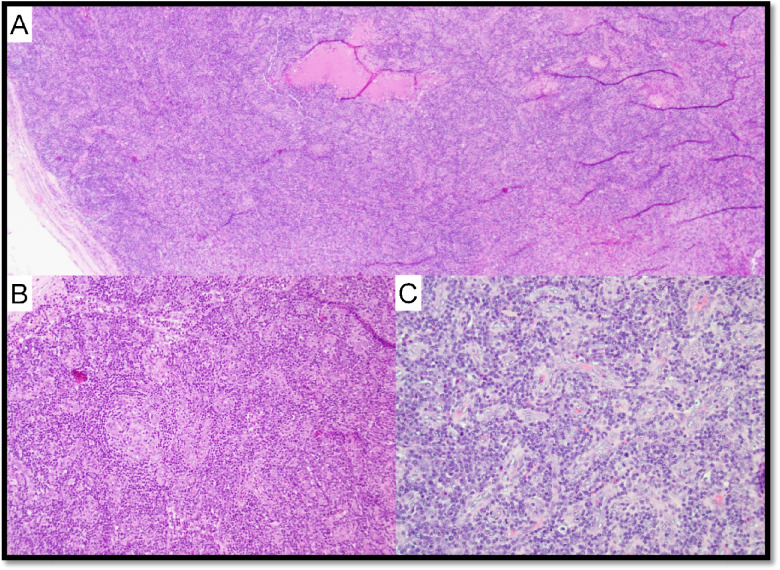
Hypervascular (HvV) morphology in TAFRO syndrome. Case LyWS-169. Courtesy of H. Shao. **A** Lymph node with respected architecture. **B** Marked vascular proliferation, atrophy of the germinal centers, and “onion layers” appearance around the follicles. **C** Marked vascular proliferation. EBV and HHV8 stainings were negative (not shown), there was no restriction of the light chains (not shown), and IgG/IgG4 ratio was below 40% (not shown)

**Fig. 8:**
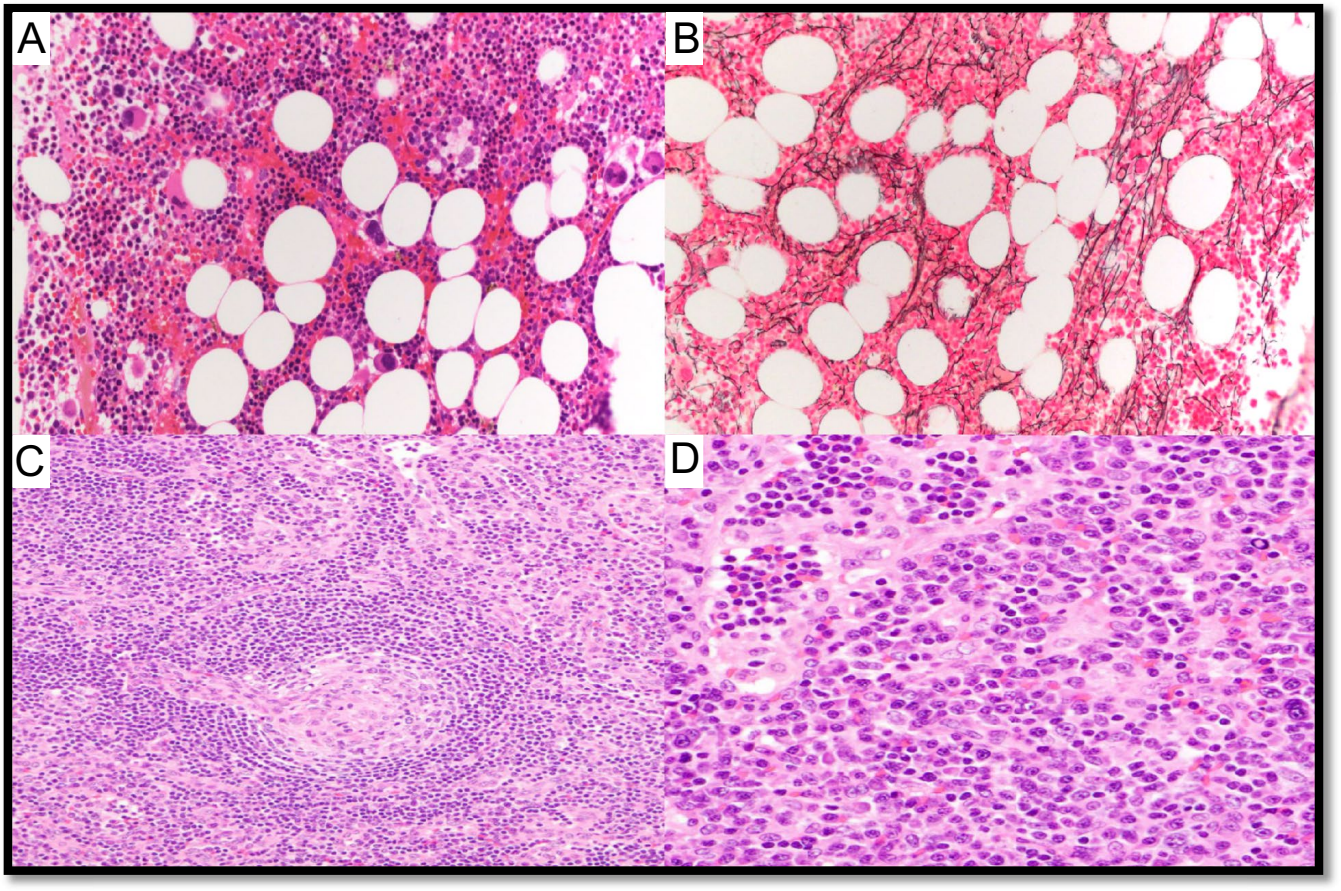
Bone marrow and lymph node changes in TAFRO syndrome. Case LyWS-153. Courtesy of M. Nichols. **A** The bone marrow shows maturing trilineage hematopoiesis with slight megakaryocytic hyperplasia without morphologic atypia. **B** Reticulin fibrosis is slightly increased (grade 1/3). **C** Lymph node changes in MCD-TAFRO: germinal centers are regressed with “lollipop follicles” and lymphocyte depletion. **D** Interfollicular plasmacytosis is present. There was no light chain restriction (not shown) and IgG/IgG4 was < 40% (not shown)

Currently, two independent sets of diagnostic criteria exist for iMCD-TAFRO syndrome (Supplementary Table 3): International criteria consider LN biopsy with CD-histopathological features as mandatory for the diagnosis (major criterium), while the Japanese TAFRO research group considers demonstration of CD-histopathology as a minor criterium, despite recommending nodal examination to exclude possible mimickers [64–66]. iMCD-TAFRO usually presents with LAD with only mildly enlarged LNs, mostly displaying HyV morphology (Fig. 7) [61, 67]. Trephine biopsies in iMCD-TAFRO show hypercellularity with megakaryocyte hyperplasia and slight atypia as well as reticulin fibrosis, thus possibly mimicking myeloproliferative neoplasms (Fig. 8). Two reports identified molecular alterations in iMCD-TAFRO patients involving *ETS1*, *PTPN6*, and *TGFBR2* genes [68, 69].

Six cases of either iMCD-TAFRO or TAFRO syndrome were submitted to the workshop, listed in Supplementary Table 4. In case LYWS-153, overlapping symptoms between iMCD-TAFRO and POEMS syndrome were present; LYWS-163 and LYWS-249 showed HyV histopathology; in LYWS-275, the diagnosis of TAFRO syndrome was rendered by multidisciplinary integration on a trephine biopsy, since the patient’s poor clinical condition prevented nodal excision, thus depicting a scenario in which Japanese TAFRO research group diagnostic criteria should be considered [66].

## CD variant of POEMS syndrome

POEMS is an acronym for a paraneoplastic syndrome characterized by polyneuropathy, organomegaly, endocrinopathy, M-protein, and skin changes, due to an underlying plasma cell neoplasm (diagnostic criteria are shown in Supplementary Table 4. In two-thirds of POEMS cases, a monoclonal plasma cell population is present in the BM biopsy, while around a third show LAD with CD-like histology (i.e., MCD-POEMS). Further common clinical presentations include papilledema, extravascular volume overload, sclerotic bone lesions, thrombocytosis/erythrocytosis, and abnormal pulmonary function tests (PEST symptoms) [70]. Similarly to other MCD subtypes, in MCD-POEMS, the clinical presentation is mostly related to the hypercytokinemia caused by the underlying plasma cell neoplasm. Cytokines proposed to drive the POEMS are VEGF, IL-6, IL-12, TGF1β, and TNF [71, 72].

Histopathologically, more than 80% of POEMS patients have BM abnormalities [70]. Megakaryocyte hyperplasia and clustering are present in 54% and 93% of cases, respectively, thus mimicking MPN [73]. Two-thirds of patients have clonal, mostly (> 90%) λ-restricted plasma cells in the BM (median BM infiltrate < 5%), half of the patients have BM lymphoid aggregates rimmed by clonal plasma cells, and one-third are diagnosed with solitary plasmacytoma [73].

**Fig. 9:**
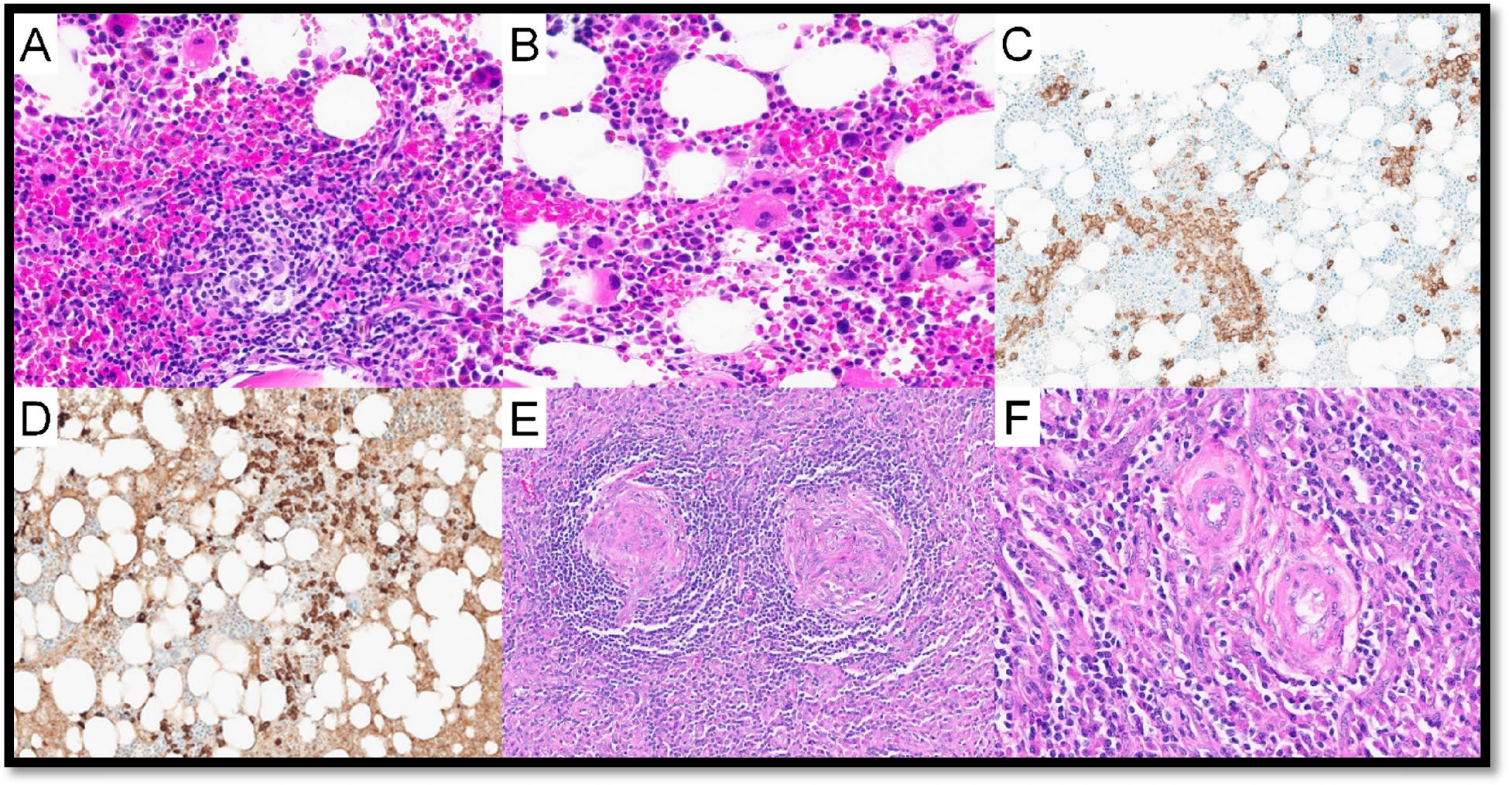
Bone marrow and lymph node changes in a patient with MCD-POEMS. Case LyWS-326. Courtesy of I. Prisneac. **A** Cellular bone marrow with a small lymphoid aggregate surrounded by plasma cells. **B** Mild megakaryocyte hyperplasia. **C** CD138 highliting rimming of the plasma cells around lymphoid aggregate. **D** Plasma cells are λ-restricted. **E** Atretic germinal centers comprised predominantly of follicular dendritic cells, surrounded by layers of mantle-type cells (onion-skinning) with “lolippop sign” and “twinning.” **F** Parafollicular regions are expanded by an increase in small blood vessels surrounded by small lymphocytes and plasma cells

Four cases of MCD-POEMS were submitted and are listed in Supplementary Table WS-5. Typical BM and LN features in MCD-POEMS are shown in Fig. 9 (LYWS-326 presented by I. Prisneac). LYWS-257 submitted by E. Hartsough represented two cases of POEMS, one worsening 6 months after SARS-CoV-2 vaccination, and the second one developed several weeks after the SARS-CoV-2 infection. So far, MCD occurring after SARS-CoV-2 infection or vaccination has been rarely reported [74]. Of interest, during SARS-CoV-2 infection, an inflammatory burst with innate immunity-mediated cytokine storm has been described and seems to be related to the severe clinical pictures [75].

## KSHV/HHV8-associated MCD

KSHV/HHV8 is a γ-herpesvirus that targets endothelial cells, B-lymphocytes, and monocytes, among others. KSHV/HHV8 encodes a viral homolog of IL-6 (vIL-6), which directly binds and activates IL-6 receptor inducing inflammation activation [76]. According to the WHO and ICC classifications, four types of frequently co-occurring and overlapping KSHV/HHV8-associated LPDs are recognized, namely primary effusion lymphoma (PEL), including extracavitary (EC-)PEL, KSHV/HHV8 + diffuse large B-cell lymphoma (KSHV/HHV8 + DLBCL), KSHV/HHV8 + germinotropic LPD (GLPD), and MCD-HHV8 [4, 77]. Their differential diagnosis is shown in Supplementary Table 5.

MCD-HHV8 is an aggressive systemic inflammatory disease typically observed in immunosuppressed individuals (either HIV + or HIV −), characterized by systemic LAD, splenomegaly, constitutional symptoms, hypergammaglobulinemia, and hypercytokinemia. Occasionally, HIV + patients present with hypercytokinemia, high KSHV/HHV8 viral load, and symptoms of systemic inflammation, but without prominent LAD: a picture is known as IL-6-related inflammatory syndrome [78].

MCD-HHV8 is histologically similar to iMCD-NOS but differs in the presence of KSHV/HHV8 + so-called “plasmablasts” in the mantle zones expressing OCT2, BLIMP1-PRDM1, IRF4-MUM1, CD38, and IgM-λ, and being negative for CD20, PAX5, CD138, and EBV. These cells are not real plasmablasts since they do not harbor somatic hypermutations as they seem to be derived from naive B-cells undergoing extrafollicular plasma cell differentiation [79]. Expansion of these “plasmablasts” with the formation of aggregates was once termed “microlymphoma,” while recently, the term was abandoned since the majority of these lesions are polyclonal and only occasionally progress to lymphoma [79, 80]. The recommended terminology is now “plasmablastic aggregates.” Other KSHV/HHV8-associated LPDs may present with a clinicopathological overlap with MCD-HHV8 leading to true diagnostic difficulties [80, 81].

Another KSHV/HHV8-associated neoplasm is Kaposi sarcoma (KS), a low-grade vascular tumor. Occasionally, focal KS may co-occur with MCD-HHV8 in the same LN. In such cases, KS typically involves the capsule, trabeculae, or LN hilus [82]. It has been hypothesized that susceptible endothelial cells are exposed to KSHV/HHV8 infection after the lysis of KSHV/HHV8-infected B-cells, resulting in the formation of local KS islets in MCD-HHV8 [83].

Altogether, 30 cases of MCD-HHV8 with or without another associated KSHV/HHV8 + LPD were submitted to the workshop (Supplementary Table WS-6). Six of them were typical MCD-HHV8; two out of the six were HIV +, three HIV −, and for one patient, HIV status was not known. All HIV + patients were older which supports the hypothesis that immune aging may play a role in increasing vulnerability to KSHV/HHV8 reactivation. In case LYWS-111, an immunocompetent patient developed MCD-HHV8 after the third SARS-CoV-2 infection.

Co-occurrence of KS with one or more KSHV/HHV8 + LPD was present in 18 out of 30 submitted cases; two-thirds of these patients were HIV +.

Seven out of 30 submitted cases showed plasmablastic aggregates in the background of MCD-HHV8. LYWS-9 submitted by S. Dirnhofer was characterized by a fulminant clinical course. In LYWS-28 (submitted by L. Xerri), a purely follicular KSHV/HHV8 +/EBV − plasmablastic proliferation was present, mimicking GLPD. Case presented by Q. Chen (LYWS-103) was a case of a 69-year-old, HIV + male with generalized LAD and pleural effusion. In the excised LN, KSHV/HHV8 + and EBV − plasmablastic population with pronounced atypia and anaplastic morphology but no architectural disruption was present. This case depicted difficulties in several differential diagnoses in the field of KHSV/HHV8 + and EBV +/− LPDs. While the overall morphologic and clinical findings were consistent with MCD-HHV8, the KSHV/HHV8 + cells were anaplastic exhibiting an unusual phenotype (CD138 +, κ/λ-, IgM/IgG-, lack all B-cell markers) not typical of KSHV/HHV8 + MCD/DLBCL but more consistent with PEL/EC-PEL. However, the extensive LAD with MCD features and lack of EBER were not characteristic of PEL. The marked morphologic atypia of KSHV/HHV8 + cells and their infiltrate in sinusoids and in paracortex were highly concerning for KSHV/HHV8 + DLBCL, but the lack of confluent sheets of large cells and overt effacement of nodal architecture made the definitive diagnosis of DLBCL difficult. Another interesting aspect of this case was that the patient had have a relatively indolent clinical course and no overt B symptoms, which was not typical of KSHV/HHV8 + DLBCL or PEL.

**Fig. 10:**
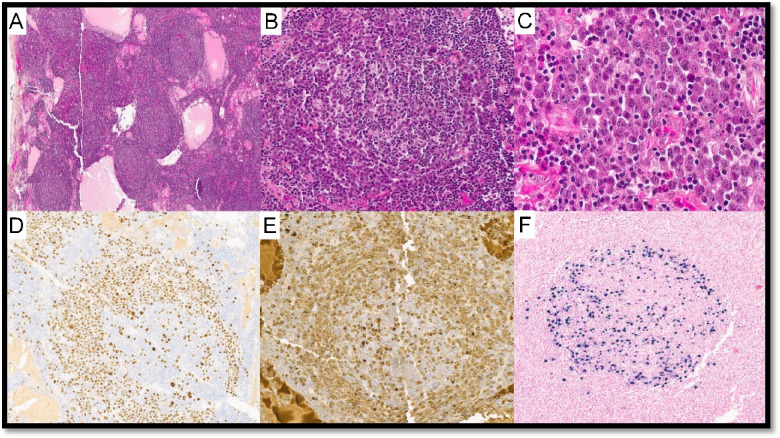
KSHV/HHV8-associated multicentric Castleman disease with plasmablast aggregates and EBV reactivation. Case LyWS-301. Courtesy of M. Donzel. **A**, **B** Lymph node architecture is preserved. The germinal centers vary in size; some have slightly “regressive” appearance. The mantle zones are thickened, sometimes with a “single file” arrangement of cells. **C** Numerous large cells with a plasmablastic appearance in aggregates are present positive for **D** HHV8 and **E** lambda, negative for CD20, CD138, and EBER (not shown). **F** Some germinal centres contain numerous EBV + cells but do not correspond to HHV8 + cells

In LYWS-301 presented by M. Donzel (Fig. 10), IGH rearrangement was found in the microdissected area but preserved LN architecture as well did not allow the diagnosis of KSHV/HHV8 + DLBCL. KSHV/HHV8 + plasmablastic aggregates can be either polyclonal or monoclonal and rarely evolve toward a KSHV/HHV8 + DLBCL with effacement of the nodal architecture [4, 79]. Recently, Rogges et al. analyzed the molecular landscape of an HIV-negative patient with KSHV/HHV8 + “microlymphoma” revealing a monoclonal rearrangement and somatic hypermutation of IGH, besides a pathogenic variant in *KMT2D* [80]. In this case, histopathology and molecular findings showed KSHV/HHV8 + LPD with features intermediate between an incipient KSHV/HHV8 + DLBCL and EBV-negative EC-PEL highlighting the challenges in the accurate classification of KSHV/HHV8-driven LPDs.

**Fig. 11:**
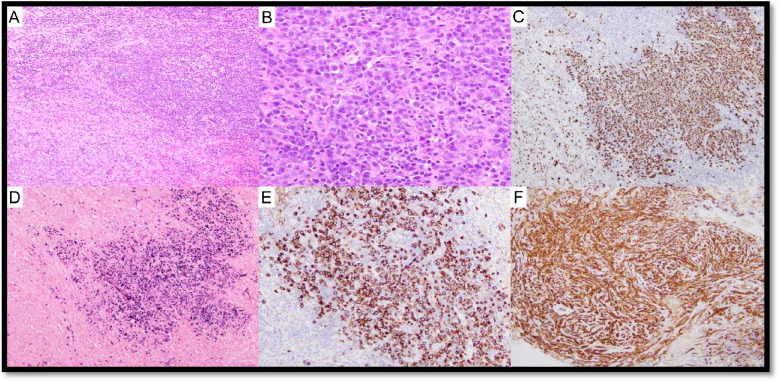
KHSV/HHV-8 and EBV-positive large B-cell lymphoma arising in the background of multicentric Castleman disease and with concurrent Kaposi sarcoma highlighting the complexity of KSHV/HHV-8-associated pathologies. Case LyWS-197. Courtesy of MG. Daniel. While WHO5 allows rare positivity for EBV in DLBCL-HHV8, panel favored the open differential diagnosis between DLBCL-HHV8 and EBV + and EC-PEL since no analysis of somatic hypermutation was performed. **A** LN with markedly distorted nodal architecture, with a prominent atypical spindle cell proliferation (lower left part)and multifocal macronodular to sheet-like aggregates of large atypical lymphoid cells (upper part).** B** Large atypical lymphoid cells have plasmablastic cytomorphology; they are positive for **C** HHV8,** D** EBER,** E** IgM, **F** spindle cell proliferation positive for CD34

Additional four cases depicted KSHV/HHV8 + and EBV +/− B-cell LPD showing complexity in their differential diagnosis and difficulties to classify all the cases in the currently defined categories. LYWS-118 by A. Y. Altay and LYWS-197 by M. G. Daniels (Fig. 11) were submitted as KSHV/HHV8 and EBV + DLBCL in the setting of MCD-HHV8. Since no analysis of somatic hypermutation was performed in either case, the panel favored an open differential diagnosis between KSHV/HHV8 and EBV + DLBCL and EC-PEL. These cases highlight the difficulties in the differential diagnosis between these two entities, despite the well-described diagnostic criteria for each entity [80]. LYWS-146 by S. Reach depicted a patient with EBV-negative PEL, while LYWS-334 by G. Frigola was a case of KSHV/HHV8 + DLBCL. Case LYWS-179 by L. Lorenzi was a GLPD with KSHV/HHV8 +/EBV + plasmablasts occupying the follicles and some sinusoidal lumen, questioning a differential diagnosis with EC-PEL. By PCR, IGH resulted in polyclonal, while RNAscope technology highlighted monotypic lambda gene expression on a fraction of atypical cells. Overall, CD138 negativity and polytypic IGH were in favor of GLPD.

In seven cases, an EBV reactivation was present. EBV + cells were either present inside or outside the GC, but in all cases did not correspond to the KSHV/HHV8 + cells. In LYWS-143 submitted by V. Tabanelli, it was proved by additionally performed double staining for EBER and HHV8 showing two different populations being positive either for EBV or HHV8. This case also raised the importance of double staining in difficult cases. How or whether KSHV/HHV8 and EBV cooperate to promote tumorigenesis remains unclear, but recent experimental data suggest that EBV/HHV8 coinfection enhances KSHV/HHV8 persistence and tumorigenesis [84, 85].

## CD-mimickers

CD-like morphological features may occur in many reactive and neoplastic diseases. Thus, to establish a formal diagnosis of CD, careful clinicopathological and laboratory correlation is mandatory [86, 87].

For the workshop, 16 cases of CD-mimickers were submitted (Supplementary Table WS-7).

### CD-like features in Lymphomas

HV-CD-like histopathological changes can be observed in different lymphomas such as follicular lymphoma (FL), mantle cell lymphoma (MCL), nodal/extranodal marginal zone lymphoma (N/EMZL), classic Hodgkin lymphoma (cHL), and nodal T follicular helper cell lymphoma, angioimmunoblastic type (nTFH, AI) [24, 88]. In addition, many clinical and laboratory features are shared between CD and lymphomas (e.g., fever, organomegaly, increased LDH and β2-microglobulin, cytopenia). Nevertheless, some changes are more specific to MCD (i.e., renal dysfunction, polyclonal hyper-γ-globulinemia, and hypoalbuminemia). Histologically, CD-like features in lymphomas are generally focal and do not involve the whole LN [89]. Such changes may either present as HV, mixed, or PV-like. Specifically in nTFH, AI pattern 1, the lymphoma itself may mimic the onion-skinning phenomenon, while in patterns 2/3, it may associate with FDC expansion occasionally mimicking the FDCS atypia of some UCD cases. Multidisciplinarity and clonality analysis may help to address the differential diagnosis [4, 90, 91].

**Fig. 12:**
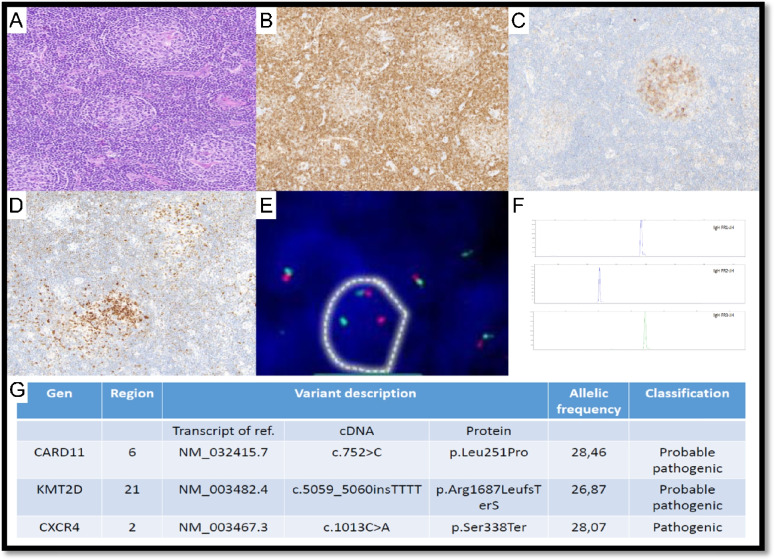
Follicular lymphoma with hyaline vascular Castleman-like features. LyWS-62. Courtesy of F. Aguirre-Neira. **A** Numerous regressed germinal centers surrounded by expanded mantle zones forming concentric rings with an “onion skin” pattern. **B** Germinal center cells are weakly BCL-2. **C** BCL-6 and **D** CD10 positive. **E** *BCL2* and *BCL6* (not shown) were rearranged by a fluorescence in situ hybridization study. **F** Clonality testing by BIOMED-2 revealed a clonal rearrangement of the IgH gene.** G** NGS showed a pathogenic variant of the *CXCR4* gene and probable pathogenic variants of the *KMT2D* and *CARD11* genes

CD-like morphology was observed in six submitted lymphoma cases: three FL (LYWS-62 submitted by F. I. Aguirre-Neira shown in Fig. 12), two MZLs, and one case of thymic cHL with CD-like features (Supplementary Table WS-7).

### Reactive lymphadenopathies

Many infections (e.g., HIV, KSHV/HHV8, CMV, EBV, syphilis, SARS-CoV-2) can induce HV-CD-like histological changes. Therefore, the differential diagnosis of CD should also include tests for infectious agents. Case LYWS-169 submitted by G. Crane depicted a rarely described case of EBV-associated reactive hyperplasia with CD-like features, progressing to cHL [92].

### Autoimmune and autoinflammatory diseases (other than IgG4-RD)

Up to 15% of LN biopsies from patients with systemic lupus erythematosus (SLE) may show histopathological features intermediate between reactive hyperplasia and HV-CD [93, 94]. In such instances, SLE should be differentiated from CD, to adopt the correct therapy. CD-like features in rheumatoid arthritis and Sjögren’s syndrome are rare [94].

A unique case of CD-like changes in Schnitzler’s syndrome (SS) was submitted (LYWS-99 by Z. Hossein-Zadeh). Histologically, SS shows a predominance of IgMk + plasma cells, while in iMCD-NOS, these are usually IgG type and polytypic [95]. LYWS-273 submitted by D. Rivera described a CD-like morphology in a patient with sarcoidosis and AA-amyloidosis. Sarcoidosis is one of the autoinflammatory conditions that can mimic iMCD-NOS, while AA-amyloidosis is rarely associated with UCD or iMCD-NOS [96–98].

### IgG4-related disease

IgG4-RD is an autoinflammatory disease of unknown etiology characterized by the presence of mass-forming lesions histologically characterized by storiform fibrosis and obliterative phlebitis together with dense lymphoplasmacytic inflammation with increased IgG4 + plasma cells (> 400/mm^2^) and IgG4 +/IgG + plasma cell ratio > 40%. Any organ can be affected, most commonly the pancreas, salivary glands, and orbit [99, 100]. Regional LAD develops in approximately half of the patients [101–103]. Five morphological patterns are described, one of which (type I) mimics iMCD [104]. The presence of an isolated increase of IgG4 + plasma cells in GCs is largely non-specific and can be observed in many reactive LADs [105]. The diagnosis of IgG4-RD can only be established on the combination of clinical and serological features together with characteristic radiological and histological appearances [102, 104].

IgG4-RD has many clinical and pathological mimickers (e.g., lymphomas, histiocytoses, autoimmune conditions) [105, 106]. As for CD, special attention is needed in iMCD, as patients with high serum IgG (> 5000 mg/dL) are likely to meet the histological diagnostic criteria for IgG4-RD [107]. However, LNs in the iMCD-NOS patients show hyperplastic GCs and interfollicular sheets of IgA +, IgG +, or IgM + plasma cells with hemosiderine deposition, while IgG4-RD shows a mixed proliferation of immature to mature plasma cells, eosinophils, small lymphocytes, and immunoblasts [108]. Clinically, CD patients are usually younger; have higher serum CRP, IL-6, IgG, IgA, IgM, and CEA levels; and present with constitutional symptoms, as opposed to IgG4-RD [105, 108]. Moreover, the serum IgG/IgG4 ratio of IgG4-RD patients is significantly higher than in CD patients. Importantly, IgG4-RD patients respond to steroid therapy which is not effective in iMCD-NOS [107–110].

Five cases of IgG4-RD LAD were submitted to the workshop, namely LYWS-106 by G. George, LYWS-312 by O. Dotsenko, LYWS-390 by T. Shet, LYWS-434 by H. Berber, and LYWS-440 by A. Ghezavati. In all cases, extensive expanded interfollicular polytypic plasma cell populations IgG4/IgG > 40% and IgG4 + plasma cells > 400/mm^2^ were present. In cases LYWS-434 and LYWS-440, storiform fibrosis was present as well, which can be a very helpful diagnostic feature.

### Others

LYWS-332 by G. Petrusevska was a case of follicular hyperplasia with some CD-like features in a patient with a possible inborn error of immunity. Case LYWS-385 by E. Dove represented a case of incidentally found nodal KS associated with CD-like changes in an immunocompetent patient.

## Conclusions

**Fig. 13:**
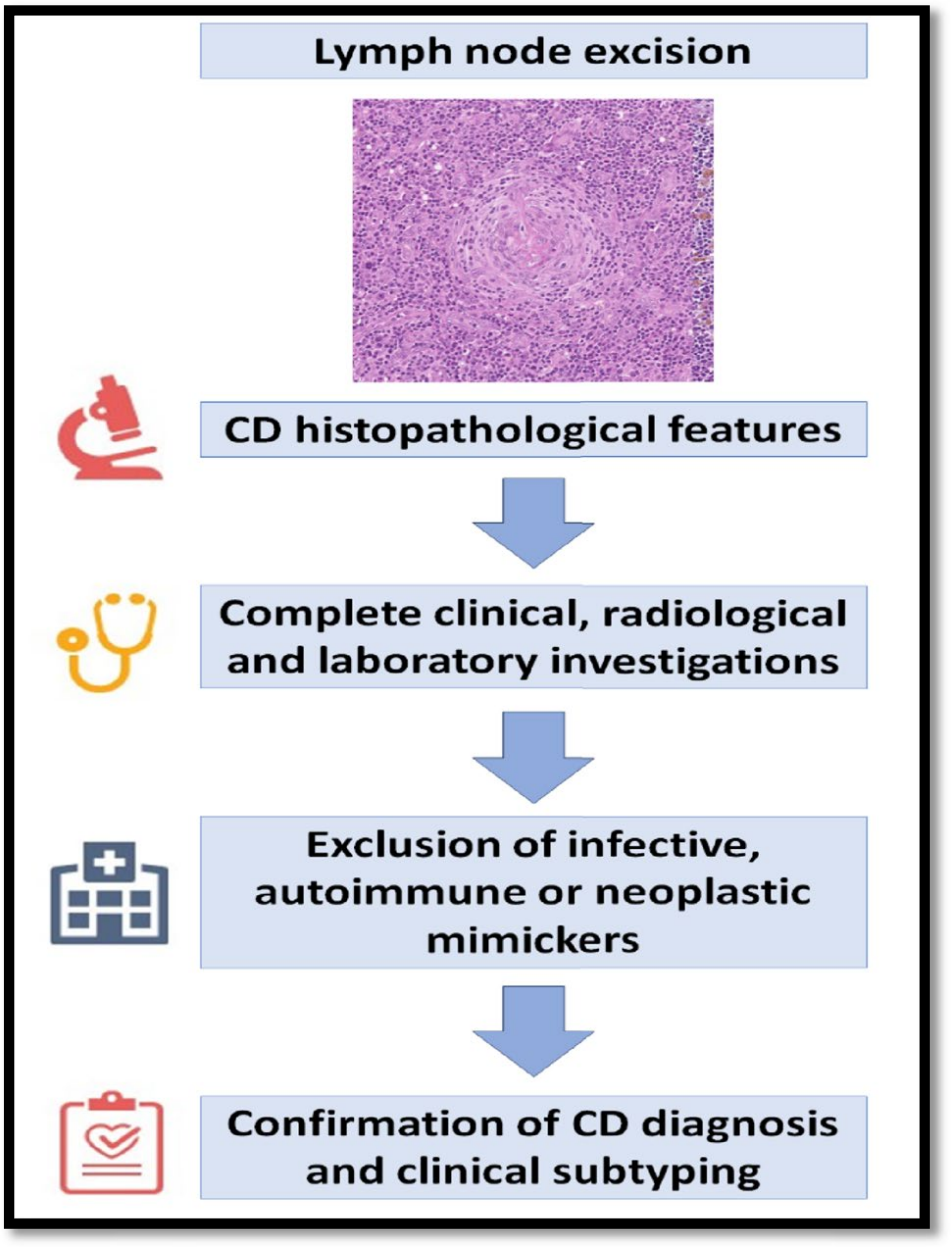
Diagnostic algorithm to diagnose Castleman disease and to exclude mimickers

The understanding of CD and the approach to its diagnosis has evolved from a histopathologically defined disease to a morphological tissue pattern in affected LNs, which can be observed in different disease entities (Box 1). In this scenario, it is nowadays clear that before making a diagnosis of CD, mimickers should be excluded based on clinical, laboratory, radiological, and morphological characteristics. Secondly, once a CD diagnosis is confirmed, it is necessary to subclassify the disease based on the multidisciplinary approach mentioned above (Fig. 13). This was well demonstrated by the cases submitted to the workshop.

**Box 1:** Major achievements on CD developed during the Lymphoma Workshop (LYWS) Session 1 at the 2024 European Association for Hematopathology (EA4HP) and Society for Hematopathology (SH) meeting in Dubrovnik

1	The understanding of CD and the approach to its diagnosis has evolved from a histopathologically defined disease to a morphological tissue pattern in affected LNs which can be observed in different disease entities. Therefore, clinical and laboratory correlation is mandatory to confirm diagnosis and exclude potential mimickers
2	HV-CD can presents with a wide morphological spectrum, ranging from predominately follicular to stromal-rich lesions. The latter is s rare variant that can represent a diagnostic challenge
3	The pathogenesis of HV CD remains enigmatic: the detection of PDGFRB-mutated cases and dysplastic stromal cells and FDCs suggest a clonal origin at least in some cases
4	Rare cases of UCD can present with systemic symptoms, relapse or progression to B-cell lymphoma
5	Rare cases of HV CD and FDCS are associated with iT-LBP
6	IMCD NOS, POEMS and TAFRO can show substantial clinical overlap; hence, strict following of defined criteria is crucial to establish the proper diagnosis
7	How TAFRO syndrome is positioned in relation to iMCD-NOS is yet to be definitively answered. Currently, there are two independent diagnostic criteria for TAFRO syndrome (i.e. International and Japanese), that differ in their approach to the major histopathological criteria
8	SARS-CoV-2 infection as well as anti-SARS-CoV-2 mRNA vaccination can induce worsening of iMCD- NOS symptoms in already diagnosed patients as well as development of iMCD-NOS, iMCD-TAFRO and CD-POEMS in rare patients
9	Cases of KSHV/HHV8+ MCD demonstrate the difficulties and overlaps in the differential diagnosis of KSHV/HHV8+ lymphoproliferative disorders. Difficulties especially exist in cases with EBV co-infection; hence, some entities in this group cannot be specifically categorized as they may present with overlapping features
10	HHV8/EBV co-infection could demand closer follow up of the patients since EBV enhances KSHV/HHV8 persistence and tumorigenesis by so far unclear mechanism

HV-CD can present with a wide morphological spectrum, ranging from predominantly follicular to stromal-rich lesions; the latter is a rare variant that can represent a diagnostic challenge. The pathogenesis of HV-CD remains enigmatic: the detection of PDGFRβ-mutated cases and dysplastic stromal cells or FDCs suggests a clonal origin at least in some cases. Dysplastic stromal cells/FDCs can be seen in HV-CD and hyperplasia–dysplasia–neoplasia model of FDC proliferation, and FDCS was proposed, but direct progression from CD has never been proven. Rare cases of UCD can present with systemic symptoms, relapse, or progression to B-cell lymphoma. As well, rare cases of HV-CD and FDCS are associated with iT-LBP.

From the cases submitted to the workshop, it was obvious that iMCD-NOS, POEMS, and TAFRO can show substantial clinical overlap; hence, strict following of defined criteria is crucial to establish the proper diagnosis. How TAFRO syndrome is positioned in relation to iMCD-NOS is yet to be definitively answered. Currently, there are two independent diagnostic criteria for TAFRO syndrome (i.e., International and Japanese) that differ in their approach to the major histopathological criteria. SARS-CoV-2 infection as well as anti-SARS-CoV-2 mRNA vaccination can induce worsening of iMCD-NOS symptoms in already diagnosed patients as well as the development of iMCD-NOS, MCD-TAFRO, and even CD-POEMS in rare patients.

Cases of KSHV/HHV8 + MCD well demonstrated the difficulties and overlaps in the differential diagnosis of KSHV/HHV8 + lymphoproliferative disorders. Difficulties especially exist in cases with EBV co-infection; hence, some entities in this group can not be specifically categorized as they may present with overlapping features. HHV8/EBV co-infection could demand closer follow-up of the patients since EBV enhances KSHV/HHV8 persistence and tumorigenesis by so far unclear mechanism.

## Supplementary Information

Below is the link to the electronic supplementary material.

ESM 1 (DOCX 81.4 KB)

## Data Availability

Not applicable.
