# Supplementary material for: The morphological spectrum of Castleman disease and related disorders: a report from the Lymphoma Workshop of the 22nd Meeting of the European Association of Hematopathology
Source: Virchows Arch. 2025 Jul 12;487(2):253–73. doi: 10.1007/s00428-025-04171-w (PMC12391218; doi:10.1007/s00428-025-04171-w)
Supplement: Supplementary file 1 — (DOCX 81.4 KB) [file 428_2025_4171_MOESM1_ESM.docx]

Suplementary Table 1. Overview of the reported genetic alterations in UCD.

| Pathways |  |
| --- | --- |
| MAPK | *FAS, PDGFRB, FGFR3, NF1,IL6ST, HRAS, KRAS, NRAS,ERBB4, JAK3, BRAF* and *TGFBR2* |
| Interleukin signaling | *PDGFRB, FGFR3, NF1, PIM1, PTPN6, IL6ST, JAK1, HRAS, KRAS, NRAS, JAK2, AKT1, ERBB4, JAK3* |
| Chromatine remodeling | None |

Adapted from A. Butzmann et al: A Review of Genetic Abnormalities in Unicentric and Multicentric Castleman Disease. Biology 2021, 10(4).

Supplementary Table 2. Summary of consensus diagnostic criteria for iMCD-NOS.

| Major criteria | Both need to be fulfilled |
| --- | --- |
| 1.Histopathologic  lymph node features | Regressed/atrophic/atretic germinal centers, often with expanded mantle zones composed of concentric rings of lymphocytes in an “onion skinning” appearance |
|  | FDC prominence |
|  | Vascularity, often with prominent endothelium in the interfollicular space and vessels penetrating into the GCs with a “lollipop” appearance |
|  | Sheetlike, polytypic plasmacytosis in the interfollicular space |
|  | Hyperplastic GCs |
| 2. Enlarged lymph nodes | (≥1 cm in short-axis diameter) in ≥2 lymph node stations |
| Minor Criteria | at least 2 of 11 criteria with at least 1 laboratory criterion need to be fulfilled |
| Clinical | 1. Constitutional symptoms: night sweats, fever (>38°C), weight loss, or fatigue (≥2 CTCAE lymphoma score for B-symptoms) |
|  | 2. Large spleen and/or liver |
|  | 3. Fluid accumulation: edema, anasarca, ascites, or pleural effusion |
|  | 4. Eruptive cherry hemangiomatosis or violaceous papules |
|  | 5. Lymphocytic interstitial pneumonitis |
| Laboratory | 1. Elevated CRP (>10 mg/L) or ESR (>15 mm/h) |
|  | 2. Anemia (hemoglobin <12.5 g/dL for males, hemoglobin <11.5 g/dL for females) |
|  | 3. Thrombocytopenia (platelet count <150 k/μL) or thrombocytosis (platelet count >400 k/μL) |
|  | 4. Hypoalbuminemia (albumin <3.5 g/dL) |
|  | 5. Renal dysfunction (eGFR <60 mL/min/1.73m2) or proteinuria (total protein 150 mg/24 h or 10 mg/100 ml) |
|  | 6. Polyclonal hypergammaglobulinemia (total γ globulin or immunoglobulin G >1700 mg/dL) |
| Exclusion Criteria | each of diseases that can mimic iMCD must be ruled out |
| Autoimmune diseases | Rheumatoid arthritis/juvenile idiopathic arthritis  IgG4-related disease  Systemic lupus erythematosus  Hemophagocytic lymphohistiocytosis/macrophage activation syndrome  Adult-onset Still disease  Autoimmune lymphoproliferative syndrome |
| Infections | Acute Epstein-Barr infection  Acute human immunodeficiency virus infection  HHV8/KSHV infection  Other infections (cytomegalovirus, tuberculosis, toxoplasmosis) |
| Malignancies | Lymphoma, Hodgkin's and non-Hodgkin's  Follicular dendritic cell sarcoma  Plasma cell neoplasms including POEMS syndrome |

Adapted from: Fajgenbaum DC et al: International, evidence-based consensus diagnostic criteria for HHV-8-negative/idiopathic multicentric Castleman disease. Blood 2017;129(12):1646-1657.

Suplementary Table 3. TAFRO Syndrome definition and criteria.

|  | International Definition of iMCD-TAFRO | 2019 Updated Criteria for TAFRO Syndrome |
| --- | --- | --- |
| Histopathology | Mandatory criteria | Minor criteria but LN biopsy strongly recommended for exclusion of mimickers |
|  | LN changes consistent with iMCD diagnostic criteria |  |
| Major criteria | All four required | All three required |
|  | Thrombocytopenia (≤100 × 10^3^/μL) | Thrombocytopenia (≤100 × 10^3^/μL) |
|  | Anasarca | Anasarca |
|  | fever (≥37.5 °C) or hyperinflammatory (CRP ≥ 2 mg/dL) status | Systemic inflammation defined as fever ≥ 37.5 °C and/or CRP ≥ 2 mg/dL |
|  | Organomegaly (small volume LAD in ≥2 regions, hepatomegaly, or splenomegaly) |  |
| Minor criteria | At least one required | At least two required |
|  | Renal insufficiency (eGFR ≤ 60 mL/min/1.73 m2, creatinine > 1 mg/dL for females and 1.3 mg/dL for males or renal failure necessitating hemodialysis) | CD-like features on LN biopsy |
|  | TAFRO-consistent BM (reticulin fibrosis or Mgc hyperplasia without evidence of an alternative diagnosis) | Reticulin myelofibrosis and/or increased number of Mgcs in BM |
|  |  | Mild organomegaly (hepatomegaly, splenomegaly, and LAD) |
|  |  | Progressive renal insufficiency |
| Supportive findings | Not required; strongly supportive | To be considered |
|  | Absence of polyclonal hypergammaglobulinemia (IgG ≤ 1.2 × normal upper limit) | Marked polyclonal hypergammopathy (IgG > 3000 mg/dL) is rare in TAFRO |
|  | Elevated ALP with mild to no elevation in bilirubin and transaminases | Obvious monoclonal protein should be absent |
|  |  | Few patients show elevated serum LDH, which indicates lymphoma. Especially intravascular large B-cell lymphoma mimics TAFRO syndrome, and random skin biopsy is recommended in such cases. |
|  |  | Most patients show elevated levels of serum ALP |
|  |  | Hepatosplenomegaly is usually mild and only confirmed by CT-scan |
|  |  | Lymphadenopathy in this disease is usually smaller than 1.5 cm in diameter |
|  |  | Exclusion criteria for Castleman disease and ITP have not been determined |
|  |  | To exclude autoimmune disorders, rheumatoid factor, anti-nuclear antibody, anti-SS-A/Ro antibody, MPO-ANCA, and PR3-ANCA have to be examined |
|  |  | To exclude of mycobacterial infections, examination of interferon-gamma release assays and ADA in pleural effusion is recommended |
|  |  | Pleural effusion and ascites in patients are usually transudative, but concentrations of IL-6 and VEGF in those fluids are usually higher than those in serum |
| Exclusion criteria | 1.Infections |  |
|  | HHV-8, EBV,acute HIV infection, TBC, COVID-19 cytokine storm syndrome | 1.Malignancies, including lymphoma, myeloma, and mesothelioma, etc.  2. Autoimmune disorders (SLE, Sjogren’s syndrome, ANCA-associated vasculitis...)  3. Infectious disorders (TBC, rickettsial disease, Lyme disease, severe fever with thrombocytopenia syndrome...)  4. POEMS syndrome  5. Hepatic cirrhosis  6. TTP/HUS |
|  | 2. Autoimmune/rheumatologic diseases |  |
|  | SLE, Sjögren syndrome, RA, adult-onset Still disease, juvenile idiopathic arthritis, IgG ≥ 3400 mg/dL (suggestive of autoimmune diseases or plasma cell dyscrasias), primary HLH |  |
|  | 3. Malignancies |  |
|  | Lymphoma, multiple myeloma, metastatic carcinoma, POEMS syndrome |  |

iMCD- idiopathic multicentric Castleman disease; LN- lymph node; CRP - C-reactive protein; LAD-lymphadenopathy; eGFR- estimated glomerular filtration rate; IgG-immunoglobulin G; LDH- lactate dehydrogenase; ALP- alkaline phosphatase; CT- computed tomography; MPO- myeloperoxidase; ANCA- anti-neutrophil cytoplasmic antibody; PR3- proteinase 3; ADA- adenosine deaminase; IL-6- interleukin-6; VEGF- vascular endothelial growth factor; ITP- immune thrombocytic purpura; HHV-8- human herpesvirus-8; EBV- Epstein–Barr virus; HIV- human immunodeficiency virus; TBC- tuberculosis; SLE-systemic lupus erythematosus; RA-rheumatoid arthritis; HLH-hemophagocytic lymphohistyocytosis; TTP-thrombotic thrombocytopenic purpura; HUS- hemolytic uremic syndrome. Adapted from Miura et al: Guidance for Managing Patients Presenting Thrombocytopenia, Anasarca, Fever, Reticulin Fibrosis, Renal Insufficiency, and Organomegaly. Biomedicines. 2024;12(6):1277.

Suplementary Table 4. Criteria for POEMS syndrome.

| Mandatory major criteria | 1. polyneuropathy (demyelinating) |
| --- | --- |
|  | 2. monoclonal plasma-cell proliferative  disorder (almost always λ) |
| Other major criteria  One required | 3. Castleman disease |
|  | 4. sclerotic bone lesions |
|  | 5.elevation of vascular endothelial  growth factor |
| Minor criteria | 6. Organomegaly (spleen, liver or lymph nodes) |
|  | 7.Extravascular volume overload (edema,  pleural effussion, ascites) |
|  | 8. Endocrinopathy (adrenal, thyroid,  pituitary, gonadal, parathyroid,  pancreatic excluding diabetes mellitus and isolated thyroid  abnormalities |
|  | 9. Skin changes (hyperpigmentation,  hypertrichosis, glomerular  hemangiomata,plethora, flushing,  white nails) |
|  | 10. Papilledema |
|  | 11. Thrombocytosis, polycythemia |
| Other symptoms | Clubbing, weigh loss, hyperhydrosis, pulmonary hypertension, restrictive lung disease, thrombotic diathesis, diarrhea, low B12 values |

The diagnosis of POEMS syndrome is confirmed when both of the mandatory major criteria, one of the three other major criteria, and one of the six minor criteria are present. Adapted from: Dispenzieri A: POEMS syndrome: Update on diagnosis, risk-stratification, and management. Am J Hematol 2023;98:1934-1950.

Suplementary Table 5. Differential diagnoses of different KSHV/HHV8+ lymphoproliferative disorders.

|  | KSHV/HHV8-MCD | PEL/EC-PEL | KSHV/HHV8-positive DLBCL | KSHV/HHV8-positive GLPD |
| --- | --- | --- | --- | --- |
| Clinical | M>F (2.2:1)  40–45 years  Majority HIV+  If HIV− patients: older frequently associated with KS | M>F  20–50 years  Immunosuppressed (HIV, posttransplant)  Frequently associated with KS or KSHV/HHV8- MCD | M>F  30–40 years  Often HIV+ patients with KSHV/HHV8-MCD | M>F  30–90 years (median: 60 years)  Most are HIV− |
| Location | LN, spleen | PEL: serous cavities  EC-PEL: LN or extranodal sites (GIT, skin, etc.) | LN, spleen, PB, extranodal sites | LN usually localized; sometimes multifocal (more common in HIV+ patients) |
| Cytomorphology | Immunoblastic/  plasmablastic | plasmablastic, immunoblastic, or anaplastic morphology | plasmablastic or immunoblastic appearance | plasmablastic, immunoblastic, or anaplastic morphology |
| Histology | Involuted to hyperplastic follicles; interfollicular vascular proliferation and plasmacytosis | Sheets of large cells effacing architecture; may be focal in LNs (EC-PEL) | Sheets of large cells effacing architecture | replacement of GCs without significant nodal architectural effacement |
| Immuno  phenotype | CD20−/+  MUM1+  IgM lambda+  vIL-6+ in majority of cells  CD138− | Few or no B-cell antigens expressed  Possible aberrant T-cell antigen expression  CD30+  MUM1+  CD138+/−  Most negative for immunoglobulin  vIL-6 variably positive | CD20+/−  MUM1+  IgM lambda+  CD138−  vIL-6+ in a large proportion of tumour cells | Usually B-cell antigen–negative  Most CD138−, CD30−; MUM1+;  immunoglobulin can be positive or negative  vIL-6 variably positive |
| KSHV/HHV8 LANA | positive | positive | positive | Positive |
| EBV (EBER) | Usually negative | Positive in>80% | Usually negative | Positive >90% |
| IG gene rearrangement and hypermutation | Polyclonal  no somatic hypermutations | Monoclonal  usually with somatic hypermutations | Monoclonal  no somatic hypermutations | Mostly polyclonal/ oligoclonal rarely monoclonal  somatic hypermutations present |

PEL- primary effusion lymphoma; EC-PEL - extracavitary primary effusion lymphoma; M-male; F-female; KS-Kaposi sarcoma; LN -lymph node; GIT-gastrointestinal tract; GC-germinal center; vIL-6 – viral interleukin 6; LANA- latency-associated nuclear antigen; EBER- EBV-encoded small RNA. Adapted from WHO 5th Ed.

**Supplementary Table WS-1. Clinicopathological features of UCD with no associated FDC proliferation**

| Case | Age | Sex | Presentation | HIV | Laboratory | Molecular studies | Unusual features | Panel diagnosis | Submitter |
| --- | --- | --- | --- | --- | --- | --- | --- | --- | --- |
| 53 | 67 y | F | Chest pain, dyspnea | NA | 0.2 g/dL IgGλ  Other normal  BM reactive | NGS: neg *BIRC3,CXCR3,KLF2,MYD88* mutations  FISH: loss of *D13S319* | UCD, PV  loss of *D13S319*: CD with clonal cytogenetic abnormality is rare, usually HV CD | UCD, PV with cytogenetic clonal aberration | E.Frye Naharro, Minneapolis, USA |
| 101* | 35 y | F | Abdominal pain, weight loss, retroperitoneal mass | neg | normal | NA | Stroma rich  iT-LBL proliferation | UCD,HV stroma-rich variant | R.King, Rochester, USA |
| 159 | 35 y | F | Enlarged retroperitoneal LN, B symptoms, | NA | ↑serum IgG and IL-6, no M-spike | NA | progression to CD5-/CD10- B cell lymphoma 5 years later  iT-LBP | UCD, mixed type, stroma-rich | S.Li, Houston,USA |
| 185 | 73 y | F | 2008 UCD, PV and MV type, right neck  2015 HV-CD,right neck. Th: siltuximab, Rituximab, ChT  2018 right cervical LAD, no B symptoms;  Right neck dissection | NA | NA | Monoclonal rearrangement of IgH in FR2 and FR3  Gene sequencing (40 gene panel): negative  FISH for t(4;14), t(11;14), t(14;16), deletion of *TP53*/monosomy  17, and deletion of 1p32/gain of 1q21: all negative | Interfollicular proliferation of monoclonal plasma cells IgA lambda  Multiple localized recurrences  Further work up for exclusion of plasma cell neoplasm was advised but patient was lost for follow up | UCD, mixed type with interfollicular proliferation of monoclonal plasma cells IgA lambda of uncertain significance | J.C.Harris, Philadelphia,  USA |
| 199 | 50 y | F | Mesenterial mass | neg | ↑serum VEGF | NA | 3x relapse over the 20 y | UCD,HV stroma-rich variant | N.Tabish, Aurora, USA |
| 217 | 49 y | F | Retroperitoneal mass, mediastinal mass | NA | Hb 8.9 g/L, other normal | No IgH rearrangement | Association with thymoma, type AB | UCD, HV associated with thymoma | D.Alquaidy, Jeddah,  Saudi Arabia |
| 224 | 36 y | F | Abdominal pain  Retroperitoneal mass | NA | NA | NA | Stroma rich | UCD,HV stroma-rich variant | M.Movassaghi, Los Angeles, USA |
| 235 | 37 y | F | Abdominal pain  Peripancreatic mass | NA | NA | NA |  | UCD, HV | V.Parecki, Portland, USA |
| 245 | 23 y | M | Back pain  Paravertebral mass D9 level | NA | NA | NA | iT-LBP | UCD, HV with iT-LBP | R.Orellana-Fernandez, Barcelona, Spain |
| 251 | 16 y | F | Cervical LAD  B symptoms | NA | NA | NA | Systemic symptoms in UCD, HV | UCD, HV | J.Reszec-Gielazyn,Bialystok, Poland |
| 261 | 59 y | M | Inguinal and pelvic LAD  No systemic symptoms  Unresectable disease- th Rituximab, no response  No evidence of polyneuropathy, endocrinopathy, skin changes | NA | IgA λ monoclonal gammopathy  BM <5%IgAλ plasma cells | No IgH rearrangement | CD, PLV with IgAλ restriction:   1. LN-based plasmacytoma? 2. Within the POEMS spectrum? | CD, PLV with IgA restricted plasma cells associated with underlying plasma cell neoplasm | O.C.Eren, New York,  USA |
| 318 | 49 y | F | Cervical LAD  History of thyroid follicular carcinoma | neg | normal | IgG and TCR rearr -*BCL1/IgH* transloc -  NGS: *SRP72* R324V mutation 51,5%VAF (VUS) | UCD, HV with probable *SRP72* germline mutation | UCD, HV with probable *SRP72* germline mutation | J.Sidhu, Binghampton,  USA |
| 362 | 39 y | F | Abdominal discomfort  Mesenterial mass | NA | No M-spike  No POEMS symptoms | NA | Perivascular/perisinusoidal plasmacytosis with lambda predominance of unknown significance | UCD, HV plasmacytosis with lambda predominance of unknown significance | R.Gulati, Indianapolis,  USA |
| 426 | 46 y | M | Back pain, B symptoms  Retroperitoneal tumor | NA | ↑ESR | IgH rearr neg | Extensive ossification | UCD, HV with extensive ossification | I.Xagoraris, Stockholm,  Sweden |
| 448 | 30 y | F | Intestinal obstruction  Mesenterial mass | NA | NA | NA |  | UCD, HV | T.Guchash, Tbilisi,  Georgia |

F-female; M-male; NA-not available; th-therapy; BM-bone marrow; NGS-next generation sequencing; FISH-fluorescent in situ hybridization; UCD-unicentric Castleman disease; PV-plasma cell variant; HV-hyaline vascular variant; MV-mixed variant; iT-LBP-indolent T lymphoblastic proliferation; ChT-chemotherapy; LAD-lymphadenopathy; VEGF-vascular endothelial growth factor; neg-negative; *case was orally presented.

**Supplementary Table WS-2. Clinicopathological features of UCD associated with atypical FDC proliferation/FDC sarcoma.**

| Case | Age | Sex | Presentation | HIV | Laboratory | Molecular studies | Unusual features | Panel diagnosis | Submitter |
| --- | --- | --- | --- | --- | --- | --- | --- | --- | --- |
| 271 | 53 y | M | Incidentally found peripancreatic mass | NA | normal | NA | Normal karyotype | FDCS arising from HV CD | L.D.Yuen, Massachusetts, USA |
| 369 | 56 y | M | Obstructive jaundice due to choledocholithiasis  Renal pelvis mass incidentally found | pos | HCV+  Other NA | IgH, IGK, TCR no rearrangement  *PDGFRA* no mutation | UCD HV in HIV+ patient  Stromal spindle cell proliferation with atypia  iT-LBP | HV CD with atypical stromal spindle cell proliferation and iT-LBP | J.Bosch-Schips,  Barcelona, Spain |
| 455 | 62 y | F | Enlarged left neck LN | NA | NA | NA | Dysplastic FDCs with loss of FDC markers  Atypical stromal cells outside GCs | HV CD with dysplastic follicular dendritic cells and loss of FDC markers | A.Green, London, UK |
| 73* | 57 y | F | Stridor, respiratory obstruction  Polypoid tracheal tumor | neg | HHV8 neg  Other NA | NA | iT-LBP  Discerning areas of HV CD, atypical FDC hyperplasia and FDCS | FDCS associated with HV CD and iT-LBP | S.Ng, Singapore, Singapore |
| 147 | 60 y | F | Paratracheal mass incidentally found | NA | NA | *TNFAIP3* K759fs*57 mutation | *TNFAIP3* K759fs*57 mutation supports association of FDCS with alterations in NF-kB pathway | FDCS arising from HV CD | Z.Hossein-Zadeh, Greenvale, USA |
| 155 | 42 y | F | Retroperitoneal mass (pelvic) incidentally found | NA | NA | NA | iT-LBP  Discerning areas of HV CD, atypical FDC hyperplasia and FDCS | FDCS arising from HV CD and iT-LBP | A.Evans, Rochester, USA |
| 205 | 57 y | F | Mesenterial mass | NA | NA | NA | Discerning areas of HV CD, atypical FDC hyperplasia and FDCS | FDCS arising from HV CD | X.N.Jiang, Shagai, China |
| 260 | 60 y | F | Right paratracheal mass | NA | NA | NA | Discerning areas of HV CD, atypical FDC hyperplasia and FDCS | FDCS arising from HV CD | O.C.Eren, New York, USA |
| 341 | 70 y | F | Epigastric pain  Perigastric tumor | NA | NA | No IgG IgK rearrangement |  | FDCS arising from HV CD | T.Tousseyn, Leuven, Belgium |

HIV-human immunodeficiency virus; M-male; F-female; NA-not available; pos-positive; neg-negative; HCV-hepatitis C virus; FDCS-follicular dendritic cell sarcoma; HV-hyaline vascular type; CD-Castleman disease.; *case was orally presented.

**Supplementary Table WS-3. Clinicopathological characteristics of patients with iMCD-NOS.**

| Case | Age | Sex | Presentation | HIV | Laboratory | Molecular studies | Unusual features | Panel diagnosis | Submitter |
| --- | --- | --- | --- | --- | --- | --- | --- | --- | --- |
| 20* | 69 y | M | Generalized LAD | neg | WBC 5600/ml  Hb 12.2 g/dL  Plt 28.8 x10^3^/ml  ↑LDH 112 U/I  ↑sIL-2R: 1000 U/ml  TP 9.4 g/dl  Alb 3.8 g/dl  ↑IgG 3485 mg/dl  ↑IgG4 802 mg/dl  IgA 347 mg/dl  IgM 88 mg/dl  ↑IL-6 8.4 pg/ml  CRP 0.87 mg/dl  ↑ESR 116 mm/h | NA | Bordeline clinicopathological features between IgG4-RD and iMCD NOS  Features favoring CD: hemosiderin-laden histiocytes, lack of eosinophils | iMCD-NOS with elevated serum IgG4 levels and abundant IgG4 + plasma cells in the LN | K.Karube, Nagoya,  Japan |
| 56 | 23 y | M | Generalized LAD, splenomegaly, constitutional symptoms, anasarca | neg | WBC 13K/μL  Hb 7.6 g/L  Plt 210 K/μL  ↑ CRP, IL-10, IL-6, sIL-2R, VEGF  Hypoalbuminemia  ↓GFR, ↑BUN, creat  Kappa BJ proteinuria  BM biopsy: reactive | No TCR, IgH, IgK rearrangement  *ATM* c.610G>A, 50% VAF (VUS)  *BRCA1* (c.341C>G, 51% VAF) | 4/5 criteria for TAFRO are fulfilled  (anasarca, fever/hyperinflammatory state, organomegaly, renal insufficiency)  Monoclonal protein in urine: suspicious for POEMS | iMCD-NOS | C.Sy, New York, USA |
| 71 | 52 y | M | Multicentric LAD | NA | ESR>145 mm/h  CRP 408 mg/L  Positive ANA  ↑IgG4 623 mg/dL  ↑IL-6 121.6 pg/mL | NA | Progression to cHL  Polyclonal plasmacytosis >40% positive for IgG4 > 100 IgG4 positive plasma cells /HPF | iMCD-NOS with increased IgG4 positive plasma cells | J.T.Kelley, Nashville,  USA |
| 402 | NA | NA | Generalized LAD  Pulmonary lesions | NA | Polyclonal hypergammaglobulinemia  ↑IgG4 11.4 g/L | NA | Polyclonal plasmacytosis >40% positive for IgG; > 100 IgG4 positive plasma cells /HPF | iMCD-NOS with increased IgG4 positive plasma cells | M.Meignin, Paris,  France |
| 439 | 46 y | M | Chronic arthralgia, generalized LAD, weight loss, fatigue | NA | Hb 113 g/L, Plt 578 K/μL,  Polyclonal hypergammaglobulinemia  ↑IL-6  BM reactive | NA |  | iMCD-NOS | K.Horvat-Pavlov, Zagreb, Croatia |

HIV-humane immunodeficiency virus; M-male; F-female; LAD-lymphadenopathy; neg-negative; NA-not available; WBC-white blood cells; Hb-hemoglobin; Plt-platelets; LDH-lactat dehidrogenase; sIL-2R-soluble interleukin-2 receptor; TP-total protein; Alb-albumin; IL-6- interleukin6; CRP-C reactive protein; ESR-erythrocyte sedimentation rate; IgG4-RD- IgG4 related disease; LN-lymph node;VEGF-vascular endothelial growth factor;GFR-glomerular filtration rate; BUN-blood urea nitrogen; BJ-Bence Jones; BM-bone marrow; ANA-antinuclear antibodies; cHL-classical Hodgkin's lymphoma; HPF-high power field; *case was orally presented.

**Supplementary Table WS-4. Clinicopathological characteristics of patients with TAFRO.**

| Case | Age | Sex | Presentation | Bone marrow  biopsy | Laboratory | Molecular studies | Unusual features | Panel diagnosis | Submitter |
| --- | --- | --- | --- | --- | --- | --- | --- | --- | --- |
| 10 | 32 y | M | Fatigue, anasarca, generalized LAD, splenomegaly, renal failure | RF gr I | ↑CRP and ESR  ↑AP  Plt 82 K/μL | NGS:  *SPEN* 48% VAF  *KMT2D* 48% VAF  *CUL4A* 53% VAF |  | iMCD-TAFRO | A.Kerper, Rochester, USA |
| 153* | 58 y | F | Demyelinating polyneuropathy  HLH criteria fullfilled  Fatigue,anasarca, renal failure, pancytopenia, fever, generalized LAD, splenomegaly  hypothyroidism | RF gr I  ↑Mgc  No plasma cell neoplasm | Pancytopenia, no details | NA | Long history of inflammatory symptoms and polyneuropathy, without improvment after corticosteroides and IVIG  1 y developing TAFRO symptoms  POEMS to be considered: polyneuropathy, hypothyroidism (but no plasma cell neoplasm) | iMCD-TAFRO | M.Nichols, Boston, USA |
| 163 | 47 y | M | Anasarca, generalized LAD, renal insufficiency | RF gr II  ↑Mgc | ↓Plt (no details) | No IgG, IgK, TCR rearrangement  NGS: negative | HyV morphology | iMCD-TAFRO | H.Shao, Tampa, USA |
| 249 | 46 y | F | Anasarca, fever, generalized LAD, hepatomegaly, multiple hematomas, renal insufficiency | No BMB; only BM aspiration –mild Mgc hyperplasia | ↓Plt 85 K/μL  ↓Hb 89 g/L  ↑creatinine 400 mmol/L | No IgG, IgK, TCR rearr. | HyV morphology | iMCD-TAFRO | J.Azacarte, Barcelona, Spain |
| 275 | 10 y | F | Prolonged febrile illness with shock  anasarca, generalized LAD, hepatosplenomegaly, | Hypercellular BM  ↑Mgc atypical  RF gr I | ↑WBC  ↓Hb  ↓Plt  No details  ↑IL-6, IL-10, TNFα | FISH: neg *BCR-ABL1* rearr.  *JAK2, CALR* neg  NGS: CNV with potential clinical significance *NOTCH1*  VUS: *CCND3, CDKN2B, KMT2A, TET2*  SNP: Gain of 9q34.3 involving part of *SEC16A* and *NOTCH1* | Dg of iMCD, TAFRO with no LN biopsy: Masaki's criteria to be considered (3 major: anasarca, ↓Plt, systemic inflammation) and 2 minor | TAFRO sy | D.Laczko, Philadelphia, USA |
| 393 | 20 y | M | Fever, anasarca, splenomegaly, generalized LAD, renal dysfunction | Hypercellular  ↑Mgc  RF gr II | ↓Plt  ↑IL-6, VEGF  Details NA | NA |  | iMCD-TAFRO | J.Xiong, Aurora, USA |

M-male; F-female; RF-reticuline fibrosis; CRP-C reactive protein; ESR-erythrocyte sedimentation rate; AP-alcaline phosphatase; Plt-platelets; NGS-next generation sequencing; VAF- variant allele frequency; HLH-hemophagocytic lymphohistiocytosis;LAD- lymphadenopathy; Mgc-megakaryocytes; IVIG-intravenous immunoglobulin; HyV-hyper vascular; BMB-bone marrow biopsy; Plt-platelets; Hb-hemoglobin; WBC-white blood cells; TNFα-tumor necrosis factor alfa; FISH-fluorescent in situ hybridization; CNV-copy number variation; SNP-single nucleotid pleomorphism; LN-lymph node;*case was orally presented.

**Supplementary Table WS-5. Clinicopathological characteristics of patients with POEMS-associated MCD**

| Case | Age | Sex | Presentation | Bone marrow biopsy | Laboratory | Molecular studies | Unusual features | Panel diagnosis | Submitter |
| --- | --- | --- | --- | --- | --- | --- | --- | --- | --- |
| 141 | 66 y | F | Generalized LAD, osteoblastic bone lesions, demyelinating polyneuropathy | ↑Mgc  Clusters of IgAλ restricted plasma cells | ↑Plt  Polyglobulia  ↑VEGF  Serum IgA λ (0.59 g/dl) | No *JAK2, CALR, MYD88* mutations  Clonal rearrangement of IgH |  | POEMS-associated MCD | L.Rodriguez Merino, Santander, Spain |
| 257 a | 65 y | F | Renal insufficiency, Raynaud phenomenon, hypothyroidism, polyneuropathy, leg edema, M-spike, generalized LAD | Plasmacytosis 10-15% IgA λ excess | ↑serum IgA λ  ↑VEGF | Negative by myeloid and lymphoid-targeted NGS panel | After Covid-19 vaccinacion | POEMS-associated MCD | E.Hartsough, Massachusetts,  USA |
| 257 b | 65 y | M | Malaise, dyspnea, peripheral neuropathy, hypothyroidism, hypogonadism, , generalized LAD, splenomegaly, skin darkening | Plasmacytosis 5-10% polytypic | ↑ serum IgA λ  Normal VEGF | Negative by myeloid and lymphoid-targeted NGS panel | After Covid-19 infection | POEMS-associated MCD | E.Hartsough, Massachusetts, USA |
| 326* | 44 y | F | Inguinal, iliacal and retroperitoneal LAD  Polyneuropathy  Skin changes, M-spike | Plasmacytosis 5-10% IgA λ | ↑VEGF  ↑serum IgA λ | IgH rearrangement in LN  BM plasma cell FISH: monosomies of chromosomes 13 & 14  Negative NGS hematological panel | Incidentally found LAD  Diagnosis of POEMS-associated MCD only after the LN biopsy | POEMS-associated MCD | I.Prisneac , Morgantown, USA |

F-female; M-male; LAD-lymphadenopathy; Mgc-megakaryocytes; Plt-platelets; VEGF-vascular endothelial growth factor; LN-lymph node; FISH-fluorescent in situ hybridization; *case was orally presented

**Supplementary Table WS-6. Clinicopathological characteristics of patients with HHV8-associated MCD**

| Case | Age | Sex | Presentation | HIV | Laboratory | Molecular studies | Unusual features | Panel diagnosis | Submitter |
| --- | --- | --- | --- | --- | --- | --- | --- | --- | --- |
| 9 | 35 y | M  Cauc. | Generalized LAD, hepatosplenomegaly,  Bicytopenia, fatigue, weight loss, myalgias | pos | Bicytopenia (details NA) | No IgH, IgK and TCR rearrang. | Aggregates of HHV8+ plasmablasts – overlapping with KHSV/HHV8+ DLBCL | KSHV/HHV8-associated MCD | S.Dirnhofer, Basel,  Switzerland |
| 28 | 50 y | M | Fever, fatigue  Generalized LAD  Hepatosplenomegaly  HLH criteria fulfilled | neg | ↓Plt, ↓Hb  ↑CRP, LDH  Polyclonal hypergammaglobulinemia  Details NA | No IgH, IgK and TCR rearr. | Purely follicular plasmablastic proliferation mimicking GLPD; EBER neg | KSHV/HHV8-associated MCD with atypical morphology | L.Xerri, Marseille, France |
| 48 | 48 y | M  Black  African  origin | Fever, weakness, weight loss, generalized LAD, hepatosplenomegaly  HLH criteria fulfilled | neg | Hb 7.1 g/dL  Plt 83 x 10^3^/L  WBC 8.4 x 10^3^/L | NA | HIV negative, immunocompetent patient | KSHV/HHV8-associated MCD with concurrent KS | F.I.Frye-Naharro, Minneapolis, USA |
| 68 | 41 y | M | Generalized LAD, pancytopenia, fatigue  HLH criteria fulfilled | pos | Pancytopenia (details NA) | NA |  | KSHV/HHV8-associated MCD with concurrent KS | T.M.Bhavsar, Washington DC, USA |
| 78 | 70 y | M | Generalized LAD, fatigue, metabolic encephalopathy | neg | NA | NA | HIV negative, immunocompetent patient | KSHV/HHV8-associated MCD with concurrent KS | J.L.Gomez-Mart, Greenvale, USA |
| 82 | 65 y | M | FUO, generalized LAD, splenomegaly | NA | Hb 7,6 g/dL  WBC 15 x 10^9^  Plt 50 x 10^9^ | NA | Splenic involvement | KSHV/HHV8-associated MCD with concurrent KS, splenic involvment | A.Bonometti, Pieve Emanuele, Italy |
| 103* | 69 y | M | Generalized LAD, pleural effusion  No B symptoms | pos | NA | *BCL6, BCL2* rearrangements neg  *IgH:MYC* fusion neg  *MYC* rearrangement present  No IgH rearrangement | Highly atypical HHV8+cells with anaplastic morphology | KSHV/HHV8-associated MCD, with proliferation of highly atypical KSHV/HHV8+ cells: KSHV/HHV8+DLBCL vs EC-PEL | Q.Chen, Chicago, USA |
| 111 | 62 y | M | FUO, B symptoms, generalized LAD, splenomegaly  HLH criteria fulfilled | neg | Ferritin 1326 ng/ml  Fibrinogen 413mg/dL  Soluble IL-2>2600 U/ml  pancytopenia | NA | HIV negative patient  Development after Covid-19 infection | KSHV/HHV8-associated MCD with associated secondary HLH | S.T.Bunting, Weston, USA |
| 118 | 49 y | M | Fever, generalized LAD, weight loss, fatigue | pos | Hb 7.5 g/dL  Plt 49 000 μL  WBC 1770 μL  ESR 97 mm/hr, CRP 31 mg/dL | NA | EBER and HHV8+ high grade B cell lymphoma: diff. Dg btw ePEL and EBER/HHV8+DLBCL | KSHV/HHV8-associated MCD with EBV+/KSHV/HHV8+ LBCL (EC-PEL vs EBV+/KSHV/HHV8+ DLBCL) since analysis of immunoglobulin somatic hypermutations was not performed | O.Altay, Istanbul, Turkey |
| 128 | 25 y | M | Fever, splenomegaly, generalized LAD, pancytopenia | pos | Pancytopenia (details NA) | NA | EBV-reactivation/non-destructive EBV-associated B cell LPD | KSHV/HHV8-associated MCD with concurrent KS and EBV reactivation | A.Samaddar, Rochester, USA |
| 143 | 22 y | M  African  origin | Fatigue, AIHA  Splenomegaly, generalized LAD | neg | ↑HHV8 DNA 3256 UI/ml  Low EBV DNA load | No IgH, IgK and TCR rearrangement  NGS lymphoma panel: negative | Aggregates of HHV8+ plasmablasts – overlapping with KHSV/HHV8+ DLBCL  EBV-reactivation/non-destructive EBV-associated B-cell LPD | KSHV/HHV8-associated MCD with EBV reactivation | V.Tabanelli, Milano, Italy |
| 146 | 86 y | M | Pleural effusion, generalized LAD, splenomegaly | pos | NA | IgK rearrangement in PE  IgH mutation rate 8.22% (>3% pos) | EBV neg PEL in HIV+ setting | KSHV/HHV8-associated MCD with subsequent PEL, EBV negative | S.Reach, New York, USA |
| 157 | 75 y | M | Generalized LAD, no B symptoms  DM, AH, prostatic adenocarcinoma | neg | NA | NA | HIV negative patient | KSHV/HHV8-associated MCD with concurrent KS | F.E.Costa, Porto, Portugal |
| 171 | 36 y | M | Fever, generalized LAD, hepatosplenomegaly, skin and mucosal lesions | pos | WBC 2.8 K/μL  Hb 7.4 g/dL  Plt 132 K/μL  LDH 673 U/L  CRP 50 mg/L  IL-6 33.6 pg/ml | NA |  | KSHV/HHV8-associated MCD with concurrent KS | H.Nabeel, New York, USA |
| 179 | 67 y | M  Cauc. | Right axillar LAD | neg | NA | No IgH rearrang.  RNAscope for light chains: monotypic lambda chain expression on fraction of atypical cells |  | KSHV/HHV8-positive germinotropic lymphoproliferative disorder with CD-like features | L.Lorenzi, Brescia, Italy |
| 197 | 53 y | M | Fever, generalized LAD, hepatosplenomegaly | pos | EBV 1.2E+0.6 IU/ml  KSHV >1.00E+08 copies/ml | Clonal IgH rearrangement  No *BCL2, BCL6,MYC, TP53* rearrangements | Rare case of EBV + KSHV/HHV8+ DLBCL  Associated KS | KSHV/HHV8-associated MCD with EBV+/HHV8+ LBCL (EC-PEL vs EBV+/HHV8+ DLBCL) and with associated KS since analysis of immunoglobulin somatic hypermutations was not performed | M.G.Daniel, New York, USA |
| 213 | 32 y | M | Generalized LAD, splenomegaly, pancytopenia, diarrhea | pos | Pancytopenia (details NA) | NA | EBV-reactivation/non-destructive EBV-associated B-cell LPD | KSHV/HHV8-associated MCD with concurrent KS and EBV reactivation | D.I.Laczko, Philadelphia, USA |
| 225 | 38 y | M  Cauc. | Known KS, generalized LAD, splenomegaly | pos | IL-6 41,87 pg/mL  CRP 240 mg/L | NA |  | KSHV/HHV8-associated MCD with concurrent KS | B.Gibson, Atlanta, USA |
| 255 | 76 y | F | Fatigue, generalized LAD, hepatosplenomegaly | NA | Anemia (details NA) | NA | EBV-reactivation/non-destructive EBV-associated B cell LPD | KSHV/HHV8-associated MCD with EBV reactivation | O.Akyol, Ankara, Turkey |
| 281 | 46 y | M | Fever, weight loss, generalized LAD, hepatosplenomegaly | pos | WBC 10.53 x 10^3^  Hb 9.2 g/dL  ↑CRP, ↑ESR  EBV DNA 461 IU/ml | No IgH, IgK and TCR rearrangements |  | KSHV/HHV8-associated MCD | F.A.Ocampo Gonzales, New York, USA |
| 282 | 30 y | M | Fever, weight loss, generalized LAD, hepatosplenomegaly | pos | WBC normal  Hb 7.5 g/dL  ↑CRP, ↑ESR  EBV DNA 364 IU/ml  HHV8 load 21200 copies/ml | NA | Dispersed TdT positive ly (iT-LBP) | KSHV/HHV8-associated MCD with concurrent KS and iT-LBP | F.A.Ocampo Gonzales, New York, USA |
| 300 | 65 y | M  Africane  origine | Fever, asthenia, weight loss, generalized LAD Cutaneous KS for 20 y | neg | NA | No IgH, IgK and TCR rearrangement |  | KSHV/HHV8-associated MCD with concurrent KS | J.Fontaine, Lyon, France |
| 301* | 75 y | M | Generalized LAD  B symptoms | neg | NA | IgH rearrangement in microdisected area | Aggregates of HHV8+ plasmablasts with no architecture effacement but IgH rearranged | KSHV/HHV8-associated MCD with plasmablast aggregates and EBV reactivation | M.Donzel, Lyon, France |
| 309 | 47 y | M | Generalized LAD  B symptoms  Cutaneous KS | pos |  | NA |  | KSHV/HHV8-associated MCD with concurrent KS and histoplasmosis | E.O.Ilori, Dallas, USA |
| 334 | 45 y | M | HHV8+MCD  Progression with generalized LAD | pos | NA | Clonal IGH rearrangement  NGS: pathogenic mutations *FAS, HRAS*; VUS mutation in *STAT5B* |  | HHV8+ DLBCL | G.Frigola, Barcelona, Spain |
| 342 | 50 y | M  Cauc. | Generalized LAD  B symptoms | neg | EBV DNA +  HHV8 DNA+  (details NA) | NA |  | KSHV/HHV8-associated MCD with EBV reactivation | M.Granai, Siena, Italy |
| 381 | 48 y | F | Generalized LAD, splenomegaly | pos | NA | NA |  | KSHV/HHV8-associated MCD with EBV reactivation | A.Saxena, Saskatoon, Canada |
| 397 | 81 y | F | Bowel obstruction | neg | NA | NA | ↑IgG4 plasma cells | KSHV/HHV8-associated MCD with increased plasma cells | N.Leone,  Siena, Italy |
| 446 | 62 y | M | Generalized LAD, fever, rash, splenomegaly  HLH criteria fulfilled | pos | Hb 4.8 g/dL  Plt 108 x 10^9^/L  WBC 2.3 x 10^9^/L | NA |  | KSHV/HHV8-associated MCD | R.Leguit, Utrecht, Netherlands |
| 452 | 32 y | M | Fever, generalized LAD, history of KS | pos | NA | NA | Aggregates of HHV8+ plasmablasts – overlapping with KHSV/HHV8+ DLBCL in CNB | KSHV/HHV8-associated MCD | A.Green, London, UK |

M-male; F-female; pos-positive; NA-not available; HLH-hemophagocytic lymphohistiocytosis; LAD-lymphadenopathy; neg-negative; Plt-platelets; Hb-hemoglobin;CRP-C reactive protein; LDH-lactate dehydrogenase; GLPD-germinotropic lymphoproliferative disorder; EBER -Epstein-Barr virus encoded small RNAs; WBC-white blood cells; KS-Kaposi sarcoma; FUO-fever of unknown origine; ESR-erythrocyte sedimentation rate; EC-PEL-extracavitary pleural effussion lymphoma; AIHA-autoimmune hemolytic anemia; LPD-lymphoproliferative disorder; PE-pleural effussion; PEL-pleural effussion lymphoma; DM-diabetes mellitus; AH-arterial hypertension; CNB-core needle biopsy.*case was orally presented.

**Supplementary Table WS-7. Clinicopathological characteristics of patients with CD-mimickers.**

| Case | Age | Sex | Presentation | Laboratory | Immunophenotype | Molecular studies | Unusual features | Panel diagnosis | Submitter |
| --- | --- | --- | --- | --- | --- | --- | --- | --- | --- |
| 62 | 70 y | M | Retroperitoneal and inguinal LAD  B symptoms | NA | CD20/BCL2/BCL6/LMO2+  CD10/HHV8/EBER-  Ki67 30% | *BCL2* and *BCL6* rearrangement  NGS: Pathogenic variant of *CXCR4* gene  Probable pathogenic variant of *CARD11* & *KMT2D* | Uncommon variant of FL | FL with HV-CD features | F.I.Aguirre Neira, Sabadell, Spain |
| 114 | 66 y | F | Axillar and right neck LAD | normal | CD20/CD10/BCL6/BCL2 +  HHV8/EBER-  Ki67 10% | *BCL2* rearrangement  NGS: VUS *ERBB2, HOXB13, TRAF2, TSC2* | Uncommon variant of FL  Long lasting SAPHO syndrome | FL with HV-CD features | G.Gasljevic, Ljubljana,  Slovenia |
| 181 | NA | F | Generalized LAD | NA | CD20/BCL6/CD23+  BCL2/EBER- | Clonal IgH rearrangement  NGS lymphoma panel: negative | Long lasting psoriatic arthritis on therapy with antirheumatic drugs and biologics | *BCL2*-R-negative CD23+ FL with HV-CD features | T.F.F.Cheng, Hong Kong, China, HKSAR |
| 273 | 62 y | F | LAD in upper abdomen, fatigue, dysphagia | NA | NA | No IgH, IgK and TCR rearrangements | Sarcoidosis with CD-like features and AA type amyloidosis | Sarcoidosis with CD-like features and AA type amyloidosis | D.Rivera, Houston,  USA |
| 258 | 38 y | M | Nasopharyngeal soft tissue mass  Cutaneous MZL 5 months previously | NA | CD20/BCL2/Kappa+  CD3/CD10/BCL6/cyclinD1/IgD/  MUM1/EBER/HHV8- | NA | Cutaneous MZL 5 months previously | EMZL with CD-HV features | S.Alshieban, Riyadh, Saudi Arabia |
| 316 | 88 y | M | Generalized LAD, no B symptoms | Plt 80000/mm^3^  Hb131g/dl  WBC 5620/mm^3^  LDH 175  IgMκ 2.7g/L | CD20/BCL2  EBER/HHV8-  Increased intrafollicular plasma cells IgG4+ (>400/HPF) IgG4/IgG>40% and κ restriction | Clonal IgH rearrangement |  | NMZL with focal CD-like features with IgG4 plasmacytic differentiation | G.Martino, Perugia, Italy |
| 325* | 69 y | F | Thymic mass | NA | CD30/CD15/PAX5 (dim) +  CD20-  EBER/HHV8 - | NA |  | Thymic cHL with CD-like features | G.Terinte-Balcan, Paris, France |
| 169 | 54 y | F | Neck LAD, intermittent fatigue; smoker  immunocompetent | NA | Variable EBV positivity by EBER with focal  reactivation in follicles or scattered paracortical cells  HHV8- | No TCR rearrangement | EBV reactive hyperplasia in immunocompetent person  CD-like features | EBV associated reactive hyperplasia with CD-like features and subsequent progression to cHL | G.Crane, Clevland Heights, USA |
| 99 | 76 y | F | Recurrent fevers, urticarial skin lesions, IgM monoclonal gammopathy  Diagnosis of Schnitzler sy 2014  Inguinal LAD | NA | Interfollicular plasma cells mostly IgM + with kappa light chain predominance  EBER/HHV8 - | Clonal rearrangement of IgH and IgK  *MYD88* L265P mutation VAF8% | LN in Schnitzler sy can morphologically mimick CD  *MYD88* mutation is unusual for CD | LN with CD-like features and clonal IgM plasmocytosis, consistent with the clinical diagnosis of Schnitzler sy | Z.Hossein-Zadeh, Greenvale, USA |
| 106 | 49 y | M | Mesenteric mass with likely involvement of adjacent small bowel loop  Hypermetabolic mesenterial LNs | ↑serum IgG4 202 mg/dL | Expanded interfollicular polytypic plasma cell population IgG4/IgG >40%  IgG4+ plasma cells> 400/mm^2^  EBER/HHV8 - | NA |  | IgG4-related LAD with CD-like features  (patterns 1,2 and 4) | G.George, Burlington, USA |
| 312 | 85 y | F | Axillary LAD | ↑serum IgG4 1.66g/L in 2021 and 2.36g/L in 2023 | Expanded intrafollicular and interfollicular polytypic plasma cell population IgG4/IgG >40%  IgG4+ plasma cells> 400/mm^2^  EBER/HHV8 - | No clonal rearrangement of IgH, IgK, TCR |  | IgG4-related LAD with CD-like features,  (patterns 1,2,3) | O.Dotsenko,  Ashford, UK |
| 390 | 59 y | M | 2018 Neck LAD  B symptoms  2020 autoimmune pancreatitis | CRP 96 IU/L  ↑serum IgG4 (details NA) | Expanded interfollicular polytypic plasma cell population IgG4/IgG >40%  IgG4+ plasma cells> 400/mm^2^  EBER/HHV8 - | IgH monoclonal peaks in polyclonal background  No TCR gene rearrangement |  | IgG4-related LAD with CD-like features,  (patterns 1,2) | T.Shet, Mumbai, India |
| 434 | 32 y | F | 12-yrs-history of RA and Graves disease  Anterior mediastinall mass | Slightely ↑ serum IgA and IgM  High CRP  RFT + | Perifollicular polytypic plasma cell population IgG4/IgG 90%  IgG4+ plasma cells> 400/mm^2^  Storiform fibrosis  EBER/HHV8 - | NA |  | IgG4-related LAD with CD-like features, pattern 1 and 5 | H.Berber, Ankara, Turkey |
| 440 | 72 y | M | Left neck mass | NA | Significant perifollicular polytypic plasma cell population IgG4/IgG >40%  IgG4+ plasma cells> 400/mm^2^  Storiform fibrosis  EBER/HHV8 - | NA |  | IgG4-related LAD with CD-like features, all patterns (admixture of features) | A.Ghezavati,  Los Angeles, USA |
| 332 | 7 y | F | PFTL previously  Generalized LAD  splenomegaly | NA | HHV8/EBER- | NA |  | FH with some CD-like features, IEI to be considered in regard to the clinical picture | G.Petrushevska, Skopje, North Macedonia |
| 385 | 69 y | F  Cauc | Multiple cancers (thyroid, breast, RCC, melanoma),retroperit. Leyomyoma  No history of ChT or RT | HIV neg | HHV8 positive KS | NA | KS in isolated LN of immunocompetent patient | Incidentally found KS in isolated LN of immunocompetent patient | E.Dove, Buffalo, USA |

IHC-immunohistochemistry; M-male; NGS-next generation sequencing; FL-follicular lymphoma; HV-hyalino vascular; CD-Castleman disease; F-female; LAD-lymhadenopathy; VUS-variant of unknown significance; SAPHO- synovitis, arthritis, pustulosis, hyperostosis, osteitis; MZL-marginal zone lymphoma; EMZL-extranodal marginal zone lymphoma; Plt-platelets; Hb-hemoglobin; WBC-white blood cells; LDH-lactate dehidrogenase; NMZL-nodal marginal zone lymphoma; cHL-classical Hodgkin's lymphoma; EBV-Epstein Barr virus; EBER- Epstein-Barr virus sy-syndrome encoded small RNAs; CRP-C reactive protein; RA-rheumatoid arthritis; RFT-rheumatoid factor test; PFTL-pediatric type follicular lymphoma; IEI-inborn error of immunity; RCC-renal cell carcinoma; ChT-chemotherapy; RT-radiotherapy.*case was orally presented.
